# Association between 24‐hour movement behaviors and adiposity in children and adolescents: A compositional data meta‐analysis

**DOI:** 10.1111/obr.13884

**Published:** 2025-01-20

**Authors:** Matthew Bourke, Zoe Harrison, Kathryn Fortnum, George Thomas, Martin O'Flaherty, Samantha K. Mulcahy, Sjaan R. Gomersall, Tahlia Alsop, Stewart G. Trost, Jennifer J. Koplin, Brianne A. Bruijns, Sophie M. Phillips, Leigh M. Vanderloo, Patricia Tucker, Kylie D. Hesketh, Matthew Y. W. Kwan, John Cairney

**Affiliations:** ^1^ Health and Wellbeing Centre for Research Innovation, School of Human Movement and Nutrition Sciences The University of Queensland Brisbane Queensland Australia; ^2^ School of Occupational Therapy, Faculty of Health Sciences Western University London ON Canada; ^3^ Australian Research Council Centre of Excellence for the Digital Child Australia; ^4^ School of Health and Rehabilitation Sciences The University of Queensland Brisbane Queensland Australia; ^5^ School of Human Movement and Nutrition Sciences The University of Queensland Brisbane Qld Australia; ^6^ Child Health Research Centre University of Queensland Brisbane Australia; ^7^ ParticipACTION Toronto Canada; ^8^ Institute for Physical Activity and Nutrition Deakin University Geelong Victoria Australia; ^9^ Department of Child and Youth Studies Brock University St. Catherines ON Canada; ^10^ INfant Child and Health Lab, Department of Family Medicine McMaster University Hamilton ON Canada

**Keywords:** adiposity, physical activity, sedentary, sleep

## Abstract

**Purpose:**

To quantitatively synthesize published evidence on the association between 24‐hour movement behavior composition with adiposity in children and adolescents aged 3–18 years.

**Methods:**

Systematic literature searches were conducted in five electronic databases to identify papers published between January 2015 and January 2024. A machine learning‐assisted systematic review was conducted to identify studies applying compositional data analysis to examine the association between 24‐hour movement behaviors and adiposity in children and youth. Random effect meta‐analyses were estimated to examine the relative association between each component of the 24‐hour movement behavior composition and body mass index *z*‐score (*z*BMI), waist circumference, fat mass percentage, and fat mass index (FMI).

**Results:**

A total of 16 studies reporting on 15,230 children and youth were included in the review. Most studies reported on *z*BMI (k = 14), followed by waist circumference (k = 5), body fat percentage (k = 3), and FMI (k = 2). Spending more time sleeping and engaged in moderate‐to‐vigorous intensity physical activity (MVPA) relative to other behaviors was associated with lower adiposity, while spending more time sedentary and engaged in light‐intensity physical activity was associated with higher adiposity.

**Conclusion:**

These results provide support for most recommendations of the 24‐hour movement behavior guidelines, including getting an adequate amount of sleep, limiting sedentary time, and engaging in MVPA, to improve adiposity outcomes.

## 1 BACKGROUND

Childhood obesity is considered a pandemic of the 21st century^1^ and is a major public health concern.^2^ Levels of overweight and obesity in children and youth increased by 47% between 1980 and 2013.^3^ It is estimated that 5% of children and adolescents worldwide currently experience obesity, equating to over 100 million children and adolescents.^4^ The rates of obesity may be even greater in many high‐income countries, including those in Western Europe (7.2% in boys and 6.4% in girls), Australasia (7.5% in boys and 7.6% in girls), and North America (12.1% in boys, 13.0% in girls).^3^ Childhood overweight and obesity are related to a range of negative health outcomes^5^, ^6^, ^7^, ^8^, ^9^ and the economic burden associated with the increased medical costs required for the management of children with obesity is estimated to be approximately $45 billion annually.^10^

The etiology of overweight and obesity is complex and is likely to be the result of an interplay between various environmental and genetic factors.^11^ Environments are now more conducive to a caloric imbalance through increased engagement in sedentary behaviors and reduced opportunities to engage in physical activity throughout the day which might contribute to childhood obesity.^12^ Additionally, not getting sufficient sleep may be linked with overweight and obesity through changes in appetite hormones that increase feelings of hunger and energy intake.^13^, ^14^ Indeed, there is strong evidence of an inverse association between physical activity and sleep duration with adiposity in children and adolescents,^15^, ^16^, ^17^ although the association with sleep may be U‐shaped.^18^ There is moderate evidence of a positive association between sedentary time and adiposity.^19^, ^20^ These findings support the implementation of 24‐hour movement behavior guidelines in early childhood through adolescence. Originally developed in Canada, 24‐hour movement behavior guidelines provide specific recommendations for levels of daily physical activity, sedentary screen time, and sleep for all children and adolescents from birth to 18 years of age.^21^ They have been adopted in a number of other countries including Australia^22^, ^23^ and South Africa^24^ and as the global guidelines by the World Health Organization (WHO) for young children aged 2–4 years.^25^

The 24‐hour movement behavior guidelines were largely developed based on the findings of the independent association between each individual movement behavior and health outcomes.^26^, ^27^, ^28^, ^29^ In the years following the introduction of the guidelines, there has been a proliferation of research examining the health benefits of achieving the overall 24‐hour movement behavior guidelines in children and adolescents. Several systematic reviews have since been conducted to examine the associations between meeting 24‐hour movement behavior guidelines and adiposity in children and adolescents.^30^, ^31^, ^32^, ^33^ Findings from these reviews generally indicate that meeting the physical activity, sedentary behavior, and sleep components of the guidelines is inversely associated with adiposity in school‐aged children and adolescents, but the association in preschool‐aged children is not as clear.

The results from the existing reviews are promising; however, the application of the findings may be limited due to a reliance on studies that simply dichotomize children into achieving and not achieving 24‐hour movement behavior guidelines. Examination of binary data in this way results in a significant loss of information,^34^ for example, disregarding the variability observed between children categorized in the same group (e.g., between children narrowly meeting guidelines vs. substantially surpassing guidelines). Additionally, dichotomizing participants disregards the underlying distribution of movement behavior variables (e.g., a child who engages in 59 minutes of MVPA each day is categorized as more like a child who engages in zero minutes of MVPA each day than a child who engages in 60 minutes of MVPA per day). The adoption of an approach that better recognizes the importance of continuous variation in multiple 24‐hour movement behaviors in contributing to adiposity is necessary and will also provide enhanced evidence on optimal cut‐point to inform future iterations of guidelines. One option is to perform compositional data analysis,^35^ which is applicable to data that carry relative rather than absolute information, including 24‐hour movement behaviors.^36^ For example, daily time spent sleeping, sedentary, engaged in LPA and MVPA are mutually exclusive, and engaging in greater levels of one of those behaviors results in less time to engage in one or more of the remaining behaviors.^37^ Therefore, the association observed between one 24‐hour movement behavior with adiposity may be in equal part the result of not spending that time engaged in another movement behavior. Adopting a compositional data analysis approach to examining 24‐hour movement behaviors can facilitate the examination of substitution effects. This can be in terms of all‐for‐one reallocations where the association between hypothetically increasing time spent in one movement behaviors at the expense of the remaining movement behaviors, or one‐for‐one reallocations where the hypothetical effect of replacing time spent in one movement behavior with time spent in a single other movement behavior is examined (this is commonly referred to as compositional isotemporal substitution modeling^38^).

In an attempt to synthesize the existing literature on 24‐hour movement behavior compositions and health, Rollo, Antsygina, Tremblay^31^ reported consistent evidence that 24‐hour movement behavior compositions were significantly related to body mass index (BMI; six studies), body composition (three studies), and waist circumference (three studies) in children and adolescents. They found that engaging in more MVPA relative to other behaviors was favorably associated with adiposity in children and adolescents. In another systematic review, authors found consistent evidence that replacing time spent sedentary, sleeping, and engaged in LPA with MVPA was associated with healthier adiposity outcomes in children and adolescents.^39^ Researchers also found consistent evidence that replacing LPA with sleep was inversely associated with adiposity.^39^ In a similar vein, in their review of studies using either compositional data analysis or isotemporal substitution modeling (c.f.,^40^) Volpato, Costa, Lopes, Sasaki, Romanzini, Ronque, Romanzini^41^ found consistent evidence that replacing sedentary time with MVPA, although not LPA, was inversely associated with adiposity in children and adolescents. Although these reviews provide an overview of the state of the evidence, the authors only narratively synthesized the existing literature, and the robustness of the results is limited by the use of vote‐counting approaches to synthesize results,^42^, ^43^ rather than estimating a pooled effect using meta‐analysis, which is almost always preferable.^44^ Using a meta‐analysis to quantify the association provides a precise effect size estimate, formally assess within‐study and between‐study variability in observed associations, and can be used to interrogate sources of heterogeneity between studies. Additionally, there has been a proliferation in the number of studies examining associations between 24‐hour movement behaviors since these previous reviews were conducted.^45^

The aim of this study was to fill the existing gap and provide quantitative estimates to improve understanding of the relationship between 24‐hour movement behavior compositions and adiposity in children and adolescents. Consistent with the rationale of the 24‐hour movement behavior guidelines, we hypothesize that the relative amount of time spent sleeping, in LPA and in MVPA will be inversely associated with adiposity outcomes and the relative amount of time spent sedentary will be positively associated with adiposity outcomes.

## 2 METHODS

This systematic review and meta‐analysis was pre‐registered on the PROSPERO register for systematic reviews (CDR: CRD42023464990) and was reported in accordance to the Preferred Reporting Items for Systematic Reviews and Meta‐Analyses.^46^

### 2.1 Study inclusion and exclusion criteria

The study inclusion and exclusion criteria in terms of participants, exposure, outcomes, and study design are outlined in Table 1.

**TABLE 1:** Inclusion and exclusion criteria.

Criteria	Inclusion	Exclusion
Participants	Children and adolescents between the ages of 3 and 18 years.	Nil.
Exposure	Assessed 24‐hour movement behaviors (self/proxy‐reported or device‐measured).	Did not report on each of the four individual parts of the 24‐hour movement behavior composition (i.e., sleep, sedentary time, LPA and MVPA).
Outcomes	Reported on a standardized and continuous measure of adiposity (e.g., BMI z‐score), waist circumference, fat‐mass‐index, percentage body fat)	Self/proxy‐reported outcomes (e.g., self‐reported height and weight)
Study designs	Cross‐sectional or longitudinal (intervention studies were eligible if they reported on associations at baseline).	Interventions; studies published before 2015; studies which are not peer reviewed (e.g., gray literature, unpublished thesis).

### 2.2 Data sources and searches

Primary literature searches were conducted on MEDLINE (via OVID), SPORTDiscus, PsycINFO, Scopus, and EMBASE from January 2015 (the year the first compositional data analysis study on 24‐hour movement behaviors was published^47^) to January 11, 2024. The search was conducted using keywords for 24‐hour movement behaviors, adiposity, and child populations (Appendix A). The search was limited to studies written in English.

### 2.3 Study selection

Identified studies were uploaded to Covidence (Veritas Health Innovation, Melbourne, Australia, https://www.covidence.org) where duplicate titles and abstracts were automatically detected and reomved. Unique titles and abstracts were uploaded to ASReview, which is an open‐source machine‐learning software used to assist in the systematic review process by semi‐automatizing the review proccess.^48^ ASReview orders titles and abstracts based on their relevance from a predicted model based on text feature of each of the records. The model is trained using prior knowledge (i.e., the labeling of relevant articles based on a preliminary search of the literature), and subsequently continuously retrained based on the reviewer's decision of the relevance of each of the articles presented by the model. Two independent reviewers completed the title and abstract screening using ASReview until the following two criteria were met: (a) 30% of all titles and abstracts were screened; and, (b) 500 consecutive titles and abstracts were labeled as irrelevant.^49^ These stopping criteria were developed based on simulation studies using ASReview which has demonstrated that when compared to manual screening of randomly ordered titles and abstracts, 100% of relevant articles are identified in the first 30% of articles screened using machine learning algorithms in reviews with greater than 5000 unique records.^48^ Given that machine learning software was used to assist the systematic review, each reviewer screened a slightly different pool of titles and abstracts. All articles that were labeled relevant by at least one reviewer were retrieved for full‐text screening and re‐uploaded to Covidence for full text screening. Each full‐text was screened by two independent authors. Where there was a disagreement between the authors, the authors discussed the article to reach a consensus decision. Where multiple studies reported on the same dataset, the study with the most complete information was included in the review, and the other studies were excluded.

### 2.4 Data extraction

Data extraction was completed by a single author and was independently checked by a second author. Data extracted included participant characteristics (e.g., age, sex, country); the method of measurement of 24‐hour movement behaviors (e.g., accelerometry, diaries); geometric means for each part of the 24‐hour movement behavior composition; the indicators used to assess adiposity; and the association between each part of the 24‐hour movement behavior composition and indicators of adiposity. To calculate the effect size for the meta‐analyses, the regression coefficient for the first log‐ratio coordinate for each behavior was also extracted. If a manuscript did not report the regression coefficient for the first log‐ratio coordinates for each behavior in the text, it was manually estimated by the lead author using the original data from the included study, applying the statistical methods described in the original paper. If the data were not publicly available, they were requested from the corresponding author. Corresponding authors were contacted on up to two separate occasions to request additional information.

### 2.5 Study quality assessment

Study quality was assessed using the Appraisal tool for Cross‐Sectional Studies (AXIS) tool.^50^ Although developed to assess the quality of cross‐sectional studies, each of the criteria is applicable to all observational research including longitudinal studies. The AXIS tool is a 20‐item tool that aims to assess the quality of cross‐sectional studies across an array of indicators, with a particular focus on the reported methods (10 items, e.g., selection bias, measurement bias, confounding) and results (5 items, e.g., reporting bias, non‐response bias). Two independent authors assessed the quality of each of the included studies. Disagreements were resolved through discussion until a consensus was achieved. Overall quality was reported as the percentage of total quality criteria that a paper received a “yes” appraisal on. Overall study quality was reported as the percentage of criteria a study received a yes appraisal on.

### 2.6 Calculation of effect sizes

The effect size used in the meta‐analyses was calculated as the absolute change in each outcome (e.g. *z*BMI) for relative changes in each part of the 24‐hour movement behavior composition compared to the remaining parts of the composition (i.e., all‐for‐one substitution). A detailed description of the method used to calculate the absolute change in each outcome for relative changes in each part of the movement behavior composition is described elsewhere.^35^ Changes in outcomes were estimated for increases in each part of the 24‐hour movement behavior composition up to 60 minutes/day, in 10‐minute increments. The standard error, 95% confidence interval, or *p*‐value, and sample size reported in the included studies were used to calculate the variance for the estimated effect sizes. Two studies reported on log‐transformed outcomes.^19^, ^51^ Results from these studies were transformed to an absolute change in the outcome to facilitate its inclusion in the meta‐analysis.^52^ Additionally, WebPlotDigitzer (Ankit Rohatgi, Pacifica, CA) was used to extract data from a single study^53^ which only reported relevant results in figures.

### 2.7 Meta‐analysis

Meta‐analyses were conducted using R v. 4.1.3 (R Core Team, Vienna, Austria) in R studio v. 1.3 (RStudio Team, Boston, MA) using the meta,^54^ metafor,^55^ and rms^56^ packages. Pooled effects were estimated using a generic inverse‐variance random‐effects meta‐analysis. A separate meta‐analysis was performed for each individual part of the 24‐hour movement behavior as the first part of the composition and each individual outcome. A meta‐analysis was conducted for any outcome that was reported in at least two studies. The restricted maximum likelihood method was used to estimate between‐study heterogeneity, which was quantified using the I^2^ statistic.

Meta‐regression was conducted for BMI *z*‐score to determine if the association between movement behavior composition was moderated by the average age of participants in each study and the quality rating of each study. Meta‐regression was not estimated for the other outcomes because they had fewer than 10 effect estimates.^57^ Meta‐regression for was estimated with restricted cubic splines to estimate the non‐linear effect of age. Models were estimated with three knots at ages 5, 10, and 15 years. Meta‐regression was based on the association between 30‐minute increase in each movement behavior.

A narrative synthesis was conducted for studies that did not report information necessary to calculate an effect size and where the study authors did not respond to a request for additional information to facilitate inclusion in the meta‐analysis.

## 3 RESULTS

### 3.1 Study selection

**FIGURE 1:**
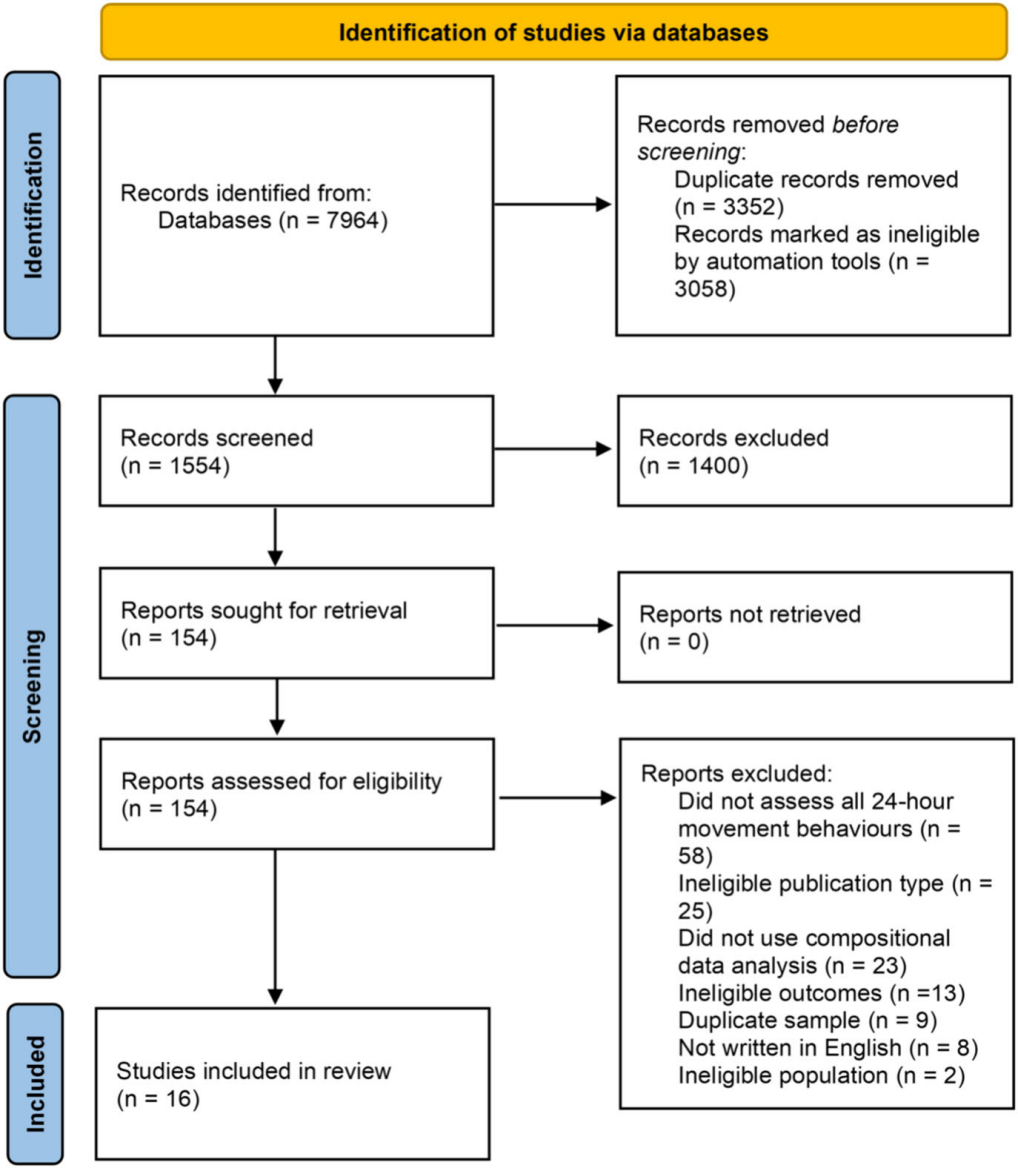
PRISMA flow diagram.

After removing duplicates, 4612 potentially relevant articles were identified. A total of 1554 titles and abstracts were examined by at least one reviewer (1215 by both reviewers, 339 by one reviewer), and the remaining 3058 titles and abstracts were ranked below the threshold for screening and were excluded. The full texts of 154 potentially relevant studies were sought for retrieval. Of these, 138 studies were excluded (see Appendix B for a full list of excluded studies with reasons) and 16 relevant studies were included in the review (see Figure 1).

### 3.2 Study characteristics

The characteristics of the included studies are reported in Table 2. Five studies were conducted in Canada,^51^, ^58^, ^59^, ^65^, ^66^ two in New Zealand,^63^, ^70^ two in China,^53^, ^60^ and a single study in each of Australia,^68^ Brazil,^61^ Czech Republic,^69^ England,^62^ Sweden,^67^ and the United States.^64^ One study reported on data from the International Study of Childhood Obesity, Lifestyle and the Environment (ISCOLE) study, an international study including data from 12 individual countries.^35^ Eight studies reported on secondary‐school‐aged children (aged 12–18 years), six studies reported on primary school‐aged children (aged 5–11 years), and three studies reported on preschool‐aged children (aged 3–4 years). All studies used accelerometers to measure sedentary time, LPA, and MVPA. Eleven studies also used sleep/wake algorithms to detect sleep duration from accelerometer data, while two studies used logbooks, two studies used parent/child questionnaires, and one study subtracted wake time from the 24‐hour period to calculate sleep. No studies reported on sedentary screen time. Studies included a range of covariates in the analysis including sex/gender (k = 13), age (k = 9), indicators of socio‐economic status (k = 9), maternal BMI (k = 3), and the consumption of unhealthy food (k = 2). Only two studies reported on longitudinal associations between 24‐hour movement behaviors and adiposity,^67^, ^70^ although only one of these studies could be included in a meta‐analysis,^70^ therefore the cross‐sectional association reported in this study was included for consistency. Funding details and declared conflicts of interests for each of the included studies are reported in Appendix C.

**TABLE 2:** Study characteristics.

Study	Country	Participants, age, and gender/sex	24‐hour movement behavior measurement (geometric mean)	Outcome measurement (arithmetic mean)	Covariates included in models
Carson et al (2016)^58^	Canada	*N* = 4169 Age = 11.4 years Sex = 48.7% female	‐ Sedentary (547 mins/day), LPA (263 mins/day), & MVPA (51 mins/day) = Actical ‐ Sleep: Self/parent‐reported average sleep (579 mins/day)	‐ BMI z‐score (WHO growth standards = 0.4) ‐ Waist‐circumference (67.2 cm)	‐ Age ‐ Sex ‐ Highest household education
Carson et al (2017)^59^	Canada	*N* = 552 Age = 3.5 years Sex = 49.2% female	‐ Sedentary (445 mins/day), LPA (230 mins/day), & MVPA (64.3 mins/day) = Actical ‐ Sleep: Self/parent‐reported average sleep (702 mins/day)	‐ BMI z‐score (WHO growth standards = 0.6) ‐ Waist‐circumference (50.6 cm)	‐ Age ‐ Sex ‐ Highest household education
Chen et al (2023)^60^	China	**Primary school students** *N* = 182 Age = 10.22 years Gender = 49.5% girls **Middle school students** *N* = 207 Age = 13.37 years Gender = 50.7% girls	‐ Sleep (518 mins/day), sedentary (570 mins/day), LPA (317 mins/day), & MVPA (35 mins/day) = ActiGraph GT3x‐BT	‐ BMI z‐score (WHO growth standards = 0.34)	‐ Gender ‐ Age
Domingues et al. (2022)^61^	Brazil	*N* = 185 Age = 15.96 years Sex = 49.2% female	‐ Sleep (494 mins/day), sedentary (727 mins/day), LPA (185 mins/day), & MVPA (34,185 mins/day) = ActiGraph GT3x	‐ BMI z‐score (WHO growth standards = 0.34)	‐ Age ‐ Sex
Dumuid et al (2018)^35^	Australia, Brazil, Canada, China, Colombia, Finland, India, Kenya, Portugal, South Africa, England, and the United States	*N* = 5828 Age = 9–11 years Gender = 54.8% girls	‐ Sleep (539 mins/day), sedentary (525 mins/day), LPA (320 mins/day), & MVPA (57 mins/day) = ActiGraph GT3x	‐ BMI z‐score (WHO growth standards = 0.45)	‐ Sex ‐ Highest parental education level ‐ Number of siblings ‐ Number of parents ‐ Study site
Fairclough et al (2017)^62^	England	*N* = 169 Age = 10.3 years Gender = 50.3% girls	‐ Sleep (549 mins/day), sedentary (510 mins/day), LPA (355 mins/day), & MVPA (26 mins/day) = ActiGraph GT9X	‐ BMI z‐score (UK reference values = 0.43) ‐ Waist‐circumference (64.3 cm) ‐ Waist‐circumference‐to‐height ratio (46%)	‐ Age ‐ Sex ‐ Indices of Multiple Deprivation decile
Haszard et al (2020)^63^	New Zealand	*N* = 742 Age = 8.0 Sex = 51.4% female	‐ Sleep (590 mins/day), sedentary (460 mins/day), LPA (320 mins/day), & MVPA (70 mins/day) = ActiGraph GT3x	‐ BMI z‐score (WHO growth standards = 0.69)	‐ No covariates
Healy et al, (2020)^64^	United States	*N* = 28 Age = 14.6 years Sex = 17.9% female	‐ Sleep (359 mins/day), sedentary (440 mins/day), LPA (576 mins/day), & MVPA (64.8 mins/day) = ActiGraph GT9X	‐ BMI (21.4)	‐ Age ‐ Sex ‐ Race
Kuzik et al (2020)^65^	Canada	*N* = 95 Age = 4.5 years Gender = 31.5% girls	‐ Sleep (666 mins/day), sedentary (366 mins/day), LPA (306 mins/day), & MVPA (108 mins/day) = ActiGraph GT3x‐BT	‐ BMI z‐score (WHO growth standards = 0.2)	‐ Home type
McGee et al, (2020)^66^	Canada	*N* = 158 Age = 5.7 years Sex = 47.0% female	‐ Sedentary (460 mins/day), LPA (242 mins/day), & MVPA (64 mins/day) = Actical ‐ Sleep (674 mins/day) = Parent completed logbooks	‐ BMI z‐score (WHO growth standards = −0.3) ‐ Body fat percentage (ADP) ‐ Fat mass index (ADP)	‐ Birth weight, ‐ Income ‐ Maternal education ‐ Duration of Breastfeeding ‐ Maternal BMI
Migueles et al, (2023)^67^	Sweden	*N* = 315 Age = 9 years Gender = NR	‐ Sleep (518 mins/day), sedentary (512 mins/day), LPA (294 mins/day), & MVPA (67 mins/day) = ActiGraph GT3x	‐ Fat‐mass‐index (ADP = 4.1 kg/m^2^)	‐ Age ‐ Sex ‐ Maternal education
Ng. et al, (2021)^68^	Australia	*N* = 1181 Age = 12.0 years Sex = 48.9% female	‐ Sleep (577 mins/day), Sedentary (560 mins/day), LPA (250 mins/day), & MVPA (53 mins/day) = GENEActiv	‐ BMI z‐score (IOTF cut‐offs = 0.44) ‐ Waist circumference (z*‐*score = 0.80) ‐ Percentage body fat (BIA (21.5)	‐ Age ‐ Sex ‐ Pubertal status ‐ Household economic position
Rasmussen et al, (2023)^69^	Czech Republic	*N* = 659 Age = 13.9 years Gender = 57.5% Girls	‐ Sleep (486 mins/day), sedentary (684 mins/day), LPA (234 mins/day), & MVPA (48 mins/day) = ActiGraph GT3x‐BT	‐ BMI z‐score (WHO growth standards = 0.22) ‐ Percentage body fat (BIA = 20.1%) ‐ Fat‐mass index (BIA = 4.3 kg/m^2^) ‐ Visceral adipose tissue (BIA = 48.8 cm^2^)	‐ Sex ‐Maternal BMI ‐ Maternal education ‐ Unhealthy snaking
Talarico et al, (2018)^51^	Canada	*N* = 458 Age = 11.5 years Sex = 50.2% female	‐ Sedentary (630 mins/day), LPA (178 mins/day), & MVPA (55 mins/day) = Actical Accelerometer ‐ Sleep (577 mins/day): Participant completed logbooks	‐ BMI z‐score (WHO growth standards) ‐ Waist‐circumference ‐Fat mass index (BIA)	‐ Age ‐ Sex ‐ Biological maturity ‐ Season of data collection ‐ Accelerometer wear time ‐ Frequency of snack food consumption in front of a screen
Taylor et al (2018)^70^	New Zealand	*N* = 248 Age = 5.0 years Sex = 49.2% female	‐ Sleep, sedentary, LPA, & MVPA = Actical Accelerometer	‐ BMI z‐score (WHO growth standards = 0.47) ‐ Percent body fat (DXA scan = 15.7%)	‐ Sex ‐ Primiparous ‐ Maternal education ‐ Household deprivation ‐ Ethnicity ‐ Maternal BMI
Zhang et al. (2022)^53^	China	*N* = 241 Age = 12.8 years Gender = 51.15 girls	‐ Sedentary, LPA, & MVPA = ActiGraph GT3x ‐ Subtracting wake time from 24 hour day	‐BMI *z*‐score	‐ Sex ‐ Age

### 3.3 Study quality

The study quality appraisal for individual studies is reported in Appendix D. Overall the total proportion of quality criteria that studies received a “yes” for ranged from 55% (11/20) to 95% (19/20), with a median of 16 (80%) criteria receiving a “yes” assessment for included studies. All studies used an appropriate study design and statistical methodologies, 15 (93.8%) reported a clear aim, discussed limitations of their study, and provided sufficient details on the study funding, ethics approval, and process to attain informed consent. Nine (56.3%) studies reported using a representative selection process to recruit participants. Seven (43.8%) reported an acceptable response rate. Only two studies (12.5%) provided a justification for their sample size.

### 3.4 Meta‐analysis

#### 3.4.1 BMI z‐score

**FIGURE 2:**
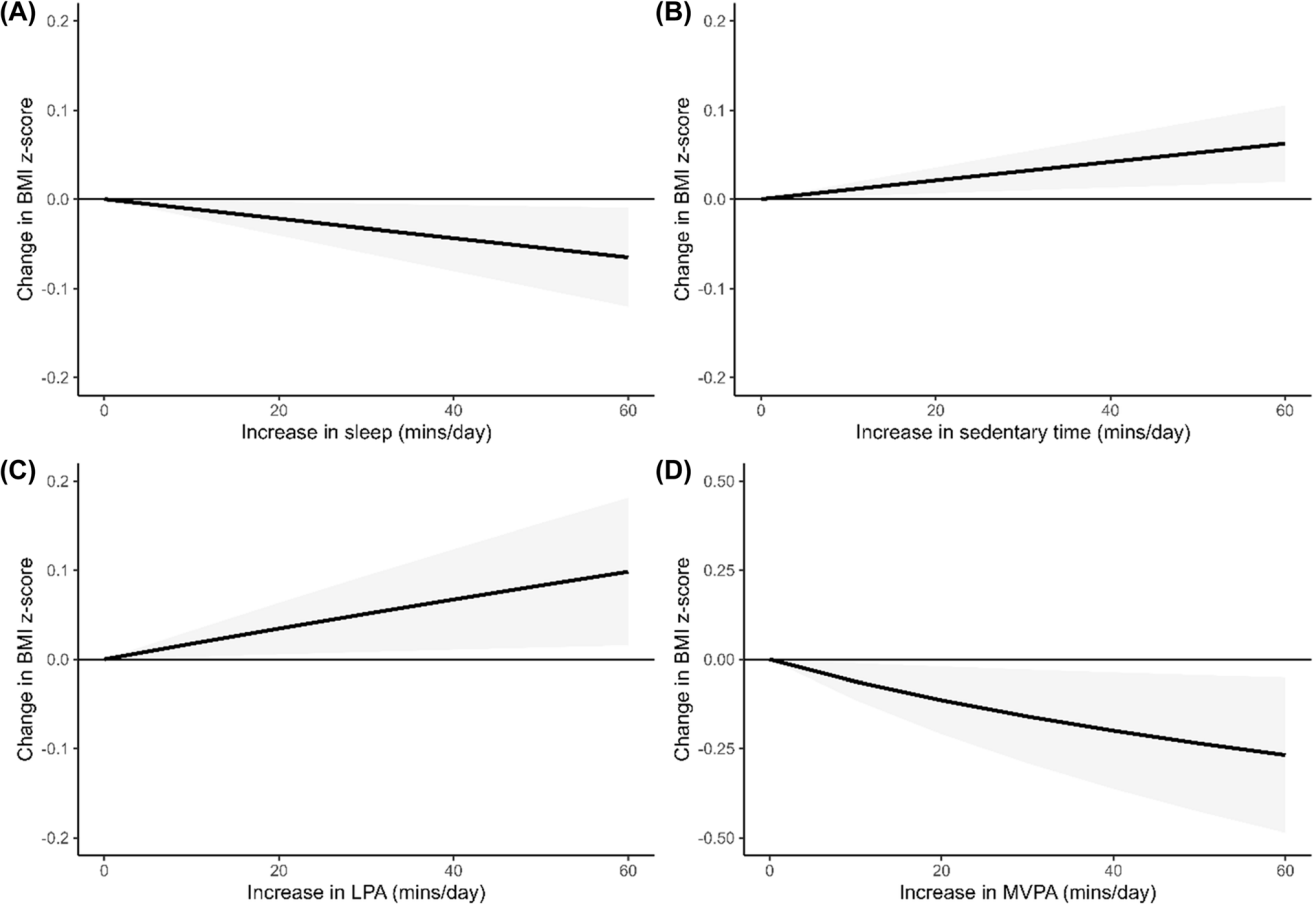
Change in zBMI associated relative changes in each part of the 24‐hour movement behavior compositions. Note that the plot for MVPA is presented on a different scale to improve readability.

A total of 14 studies,^35^, ^51^, ^53^, ^58^, ^59^, ^60^, ^61^, ^62^, ^63^, ^65^, ^66^, ^68^, ^69^, ^70^ reporting on 15 unique effect sizes and including 15,011 participants were included in the meta‐analyses for BMI *z*‐score. The results from the meta‐analyses are presented in Figure 2. The amount of time spent sleeping relative to the other 24‐hour movement behaviors was inversely associated with BMI *z*‐score (*z* = −2.30, *p* = 0.021, I^2^ = 79.1%; Figure 2a). The amount of time spent engaged in MVPA relative to the other 24‐hour movement behaviors was also inversely related to BMI *z*‐score (*z* = −2.39, *p* = 0.017, I^2^ = 89.7%; Figure 2d). Conversely, time spent sedentary relative to other movement behaviors (*z* = 2.87, *p* = 0.004, I^2^ = 61.8%; Figure 2b) and time spent engaged in LPA relative to other 24‐hour movement behaviors (*z* = 2.34, *p* = 0.019, I^2^ = 89.7%; Figure 2c), were related to higher BMI *z*‐scores.

**FIGURE 3:**
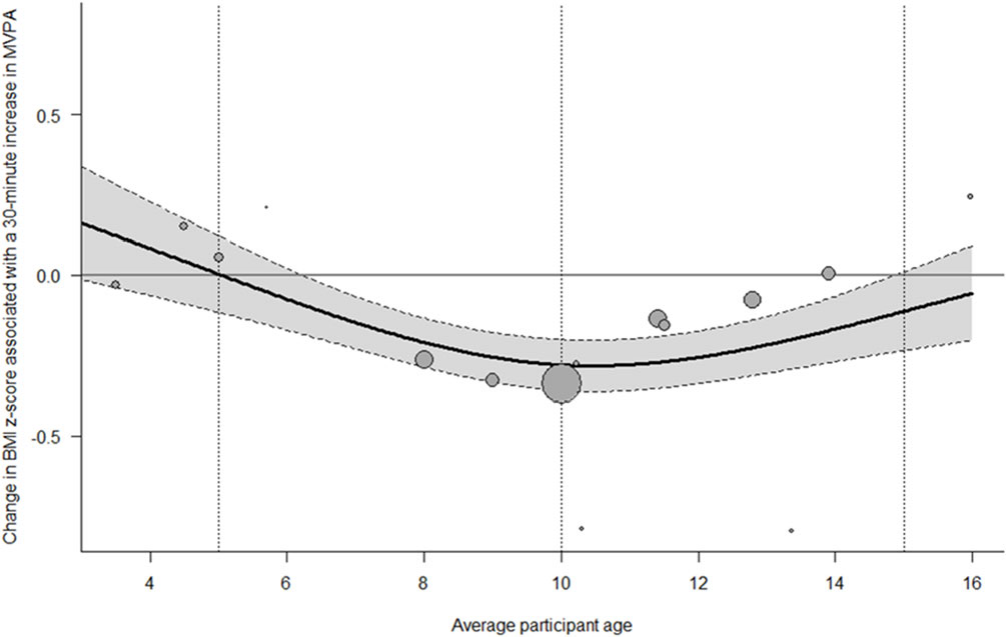
Bubble plot of the results from the meta‐regression of the association between time spent in MVPA relative to other 24‐hour movement behaviors on BMI z‐score regressed on average participant age.

Meta‐regression analyses were conducted to determine if the association between components of the 24‐hour movement behavior composition and BMI *z*‐score differed based on the average age of participants included in each study and the quality rating of each study. Results showed that the average age of participants moderated the association between the amount of time spent engaged in MVPA relative to other 24‐hour movement behaviors and BMI *z*‐score (Q = 16.56, *df* = 2, *p* < 0.001; Figure 3). Among children and adolescents between 6 and 13 years, there were significant inverse associations between MVPA and BMI *z*‐score, while the association was not significant for children younger than 6 years or older than 13 years. There was no significant difference in association between time spent sleeping (Q = 3.18, *df* = 2, *p* = 0.204), being sedentary (Q = 2.77, *df* = 2, *p* = 0.251), or engaging in LPA (Q = 1.66, *df* = 2, *p* = 0.436) with BMI *z*‐score as a function of participants age. Additionally, the association between 24‐hour movement behaviors and BMI *z*‐score did not differ as a function of the study quality rating (*p* > 0.05).

#### 3.4.2 Waist circumference

**FIGURE 4:**
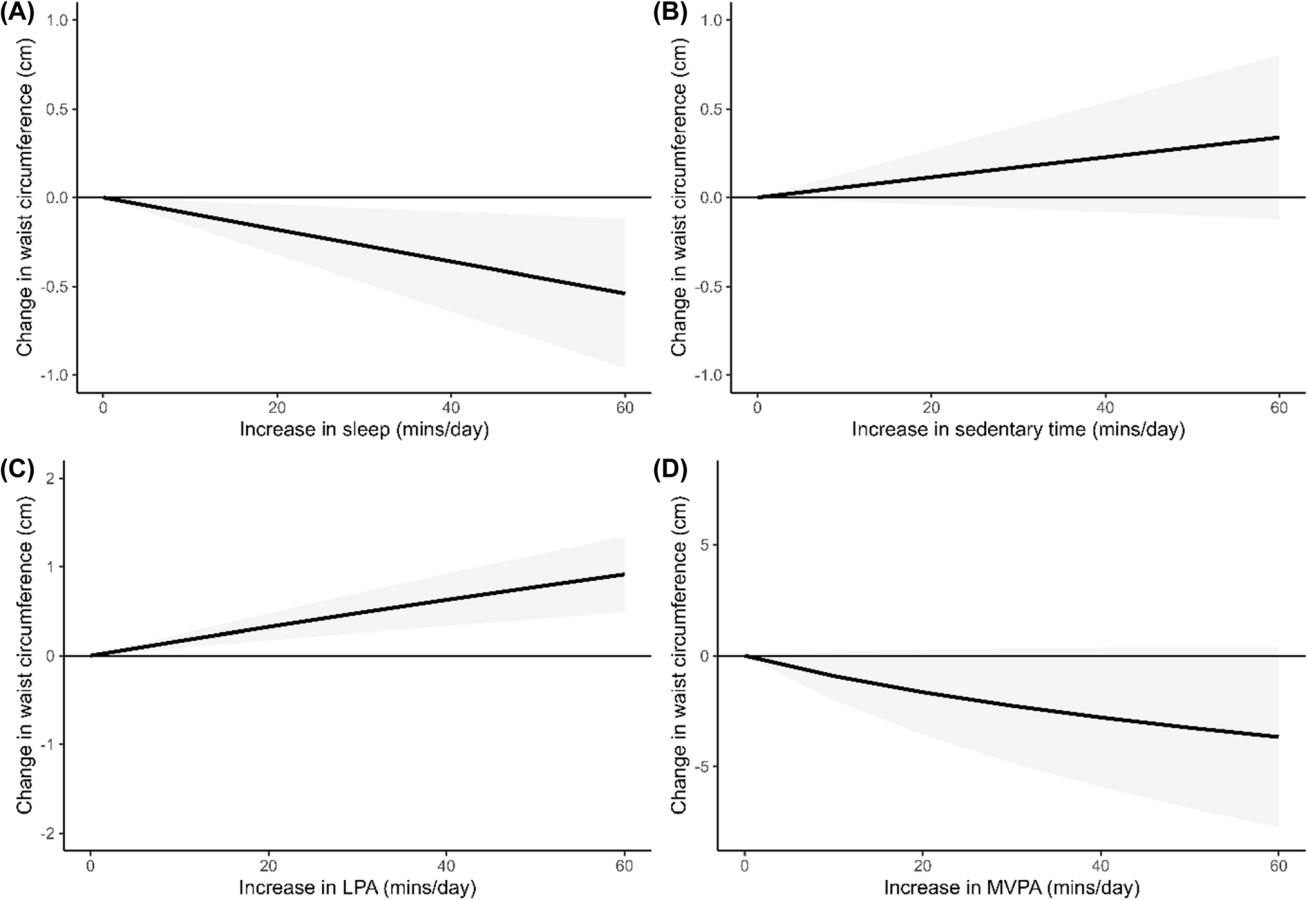
Change in waist circumference associated with relative changes in each part of the 24‐hour movement behavior composition. Note that the plots are presented on a different scale to improve readability.

Five studies,^51^, ^58^, ^59^, ^62^, ^68^ reporting on 5078 participants were included in the meta‐analysis for waist circumference. Results demonstrated spending more time sleeping (*z = ‐2.51, p* = 0.012, I^2^ = 42%; Figure 4a) relative to other 24‐hour movement behaviors was inversely associated with waist circumference. Spending more time engaged in LPA relative to other 24‐hour movement behaviors was associated with larger waist circumference (z = 2.29, *p* = 0.022, I^2^ = 50%; Figure 4c). Time spent sedentary (z = 1.44, *p* = 0.149, I^2^ = 55%; Figure 4b) and time spent engaged in MVPA (z = ‐1.72, *p* = 0.086, I^2^ = 95%; Figure 4d) did not have a significant association with waist circumference. Results of the meta‐analysis for waist‐circumference are presented in Figure 4.

#### 3.4.3 Percentage body fat

**FIGURE 5:**
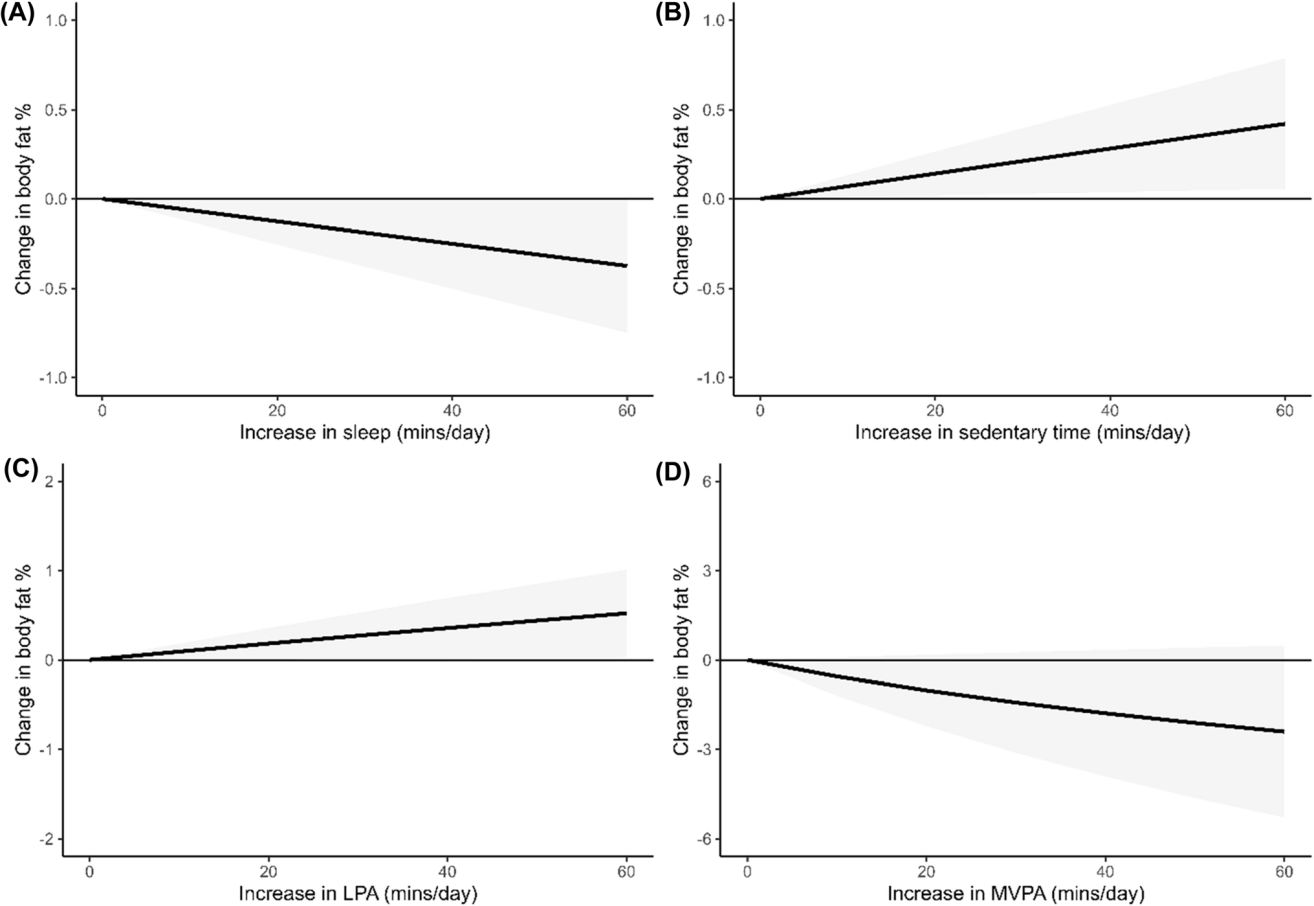
Change in body fat percentage associated with relative changes in each part of the 24‐hour movement behavior composition. Note that the plots are presented on a different scale to improve readability.

Three studies,^68^, ^69^, ^70^ reporting on 2053 participants were included in the meta‐analysis for body fat percentage. Results showed that time spent sleeping relative to other 24‐hour movement behaviors was associated with lower body fat percentage (*z* = −1.95, *p* = 0.051, I^2^ = 0.0%; Figure 5a). Time spent sedentary (*z* = 2.26, *p* = 0.024, I^2^ = 0.0%; Figure 5b) and LPA (*z* = 2.08, *p* = 0.037, I^2^ = 29.2%; Figure 5c) relative to other movement behaviors was associated with higher body fat percentage. The association between time spent engaged in MVPA (*z* = −1.66, *p* = 0.097, I^2^ = 92.1%; Figure 5d) relative to other 24‐hour movement behaviors with body fat percentage was not statistically significant. An overview of the association between the relative association between each part of the 24‐hour movement behavior composition and body fat percentage is displayed in Figure 5.

#### 3.4.4 Fat‐mass‐index

**FIGURE 6:**
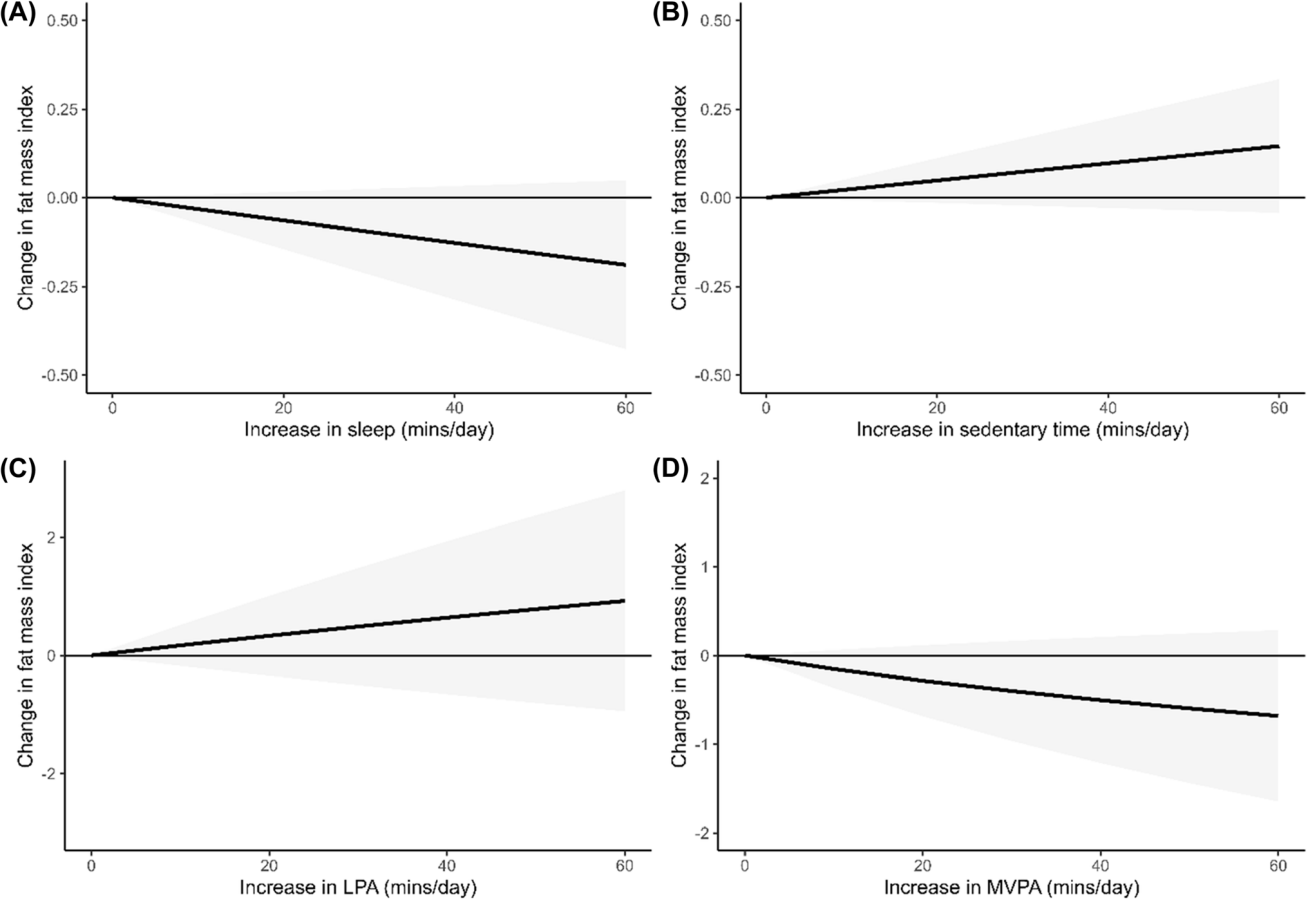
Change in fat mass index associated with relative changes in each part of the 24‐hour movement behavior composition. Note that the plots are presented on a different scale to improve readability.

Two studies^51^, ^69^ including 1093 participants were included in the meta‐analysis for FMI. Although the results followed the same pattern as other outcomes, results from the meta‐analysis found no statistically significant associations between relative time spent sleeping (*z* = −1.56, *p* = 0.119, I^2^ = 0.0%; Figure 6a), sedentary (*z* = 1.52, *p* = 0.130, I^2^ = 0.0%; Figure 6b), engaged in LPA (*z* = 0.97, *p* = 0.333, I^2^ = 85.5%; Figure 6c) or MVPA (*z* = −1.39, *p* = 0.165, I^2^ = 81.5%; Figure 6d) with FMI. The results from the meta‐analyses with FMI are displayed in Figure 6.

### 3.5 Overview of studies not included in meta‐analyses

Three studies did not provide sufficient information to be included in the meta‐analyses. One study reported on BMI in 7–19‐year‐old autistic children,^64^ but did not provide results for BMI *z*‐scores, so could not be included in the meta‐analysis. Results from that study demonstrated that spending more time engaged in LPA and sedentary time relative to other 24‐hour movement behaviors was associated with higher BMI and spending more time sleeping relative to other 24‐hour movement behaviors was associated with lower BMI. Time in MVPA relative to other 24‐hour movement behaviors was not significantly related with BMI. An additional two studies could not be included in the meta‐analysis because the authors did not report enough information to calculate an effect size and did not respond to emails for additional information.^66^, ^67^ One of these demonstrated that no parts of children's 24‐hour movement behavior relative to other behaviors were significantly related to either percentage body fat or FMI in 5‐year‐old children.^66^ Results from the other of these studies showed that time spent engaged in MVPA relative to other 24‐hour movement behaviors was inversely associated with FMI cross‐sectionally in 9‐year‐old children, and more sedentary time relative to time spent sleeping was associated with higher FMI.^67^ However, 24‐hour movement behaviors at age 4 were not longitudinally related to FMI at age 9 in that study.

## 4 DISCUSSION

The aim of this systematic review and meta‐analyses was to synthesize research on the association between children and adolescent's 24‐hour movement behavior composition and adiposity‐related outcomes. To our knowledge, this study is the first to quantitatively synthesize results from multiple studies using compositional data analysis to examine the association between 24‐hour movement behaviors and adiposity. These results extend upon previous observational research on the association between individual components of 24‐hour movement behavior compositions and adiposity by accounting for the interdependencies between each of the 24‐hour movement behaviors and reflecting the reality of a constrained 24‐hour time use budget. A total of 16 studies that met most of the assessed quality criteria were identified and included in meta‐analyses examining the association between 24‐hour movement behavior compositions and BMI z‐score, waist circumference, percentage body fat, and fat‐mass index. Results from the meta‐analyses provided consistent evidence that spending more time sleeping and engaged in MVPA relative to other 24‐hour movement behaviors are associated with lower adiposity, whereas time spent sedentary and engaged in LPA relative to other 24‐hour movement behaviors is associated with higher adiposity during childhood and adolescence. The strongest association with each outcome was observed for time spent in MVPA relative to other movement behaviors. For example, spending 60 more minutes in MVPA each day relative to other movement behaviors was associated with a 0.27 unit lower BMI *z*‐score, an association that is widely considered clinically meaningful.^71^ A similarly sized association with BMI *z*‐score in a favorable direction was observed at much higher levels of sleep relative to other behaviors (4 hours/day), and in an unfavorable direction for the amount of time spent sedentary (4 hours/day) and in LPA (3 hours/day) relative to other behaviors. By and large, the results from the current study are in accordance with studies that have examined the association between achieving 24‐hour movement guidelines and adiposity,^30^, ^31^, ^32^, ^33^ and support the general message of 24‐hour movement behavior guidelines, with regards to children and adolescents getting an adequate amount of sleep, limiting the amount of time spent sedentary, and maximizing the amount of time they spend in MVPA.

Interestingly, results from the current meta‐analyses showed that time spent in LPA relative to other behaviors was associated with higher adiposity. Similar results were previously reported in a meta‐analysis of studies using isotemporal substitution modeling to examine the association of replacing sedentary time with LPA and MVPA, which found that only replacing sedentary time with MVPA, but not LPA, was inversely related with body fat percentage.^72^ Results in a separate systematic review also found that only replacing sedentary time with MVPA, but not LPA, was consistently inversely associated with BMI in children and adolescents.^41^ Although the one‐for‐one substitution effect was not examined in the present study, similarities in the magnitude of the association between relative time spent sedentary and in LPA with each of the examined outcomes indicate that the substitution effects between sedentary time and LPA would likely be small. Therefore, the benefits of engaging in several hours of LPA each day in children may be limited, at least with respect to weight status or adiposity. It is important to note that these findings do not indicate that higher levels of LPA are directly associated with adiposity, per se, but instead may indicate that children who spend large amounts of time engaged in LPA may be doing so at the expense of other movement behaviors which are more strongly inversely associated with adiposity. These results may also be a consequence of how LPA is measured. Although accelerometery is generally considered best practice for assessing physical activity in youth, the cut point method used to predict physical activity intensity in the included studies consistently misclassify LPA as either MVPA or sedentary time, while also misclassifying sedentary time and MVPA as LPA.^73^, ^74^ Implementing more advanced accelerometer data processing approaches which consider posture and acceleration and leveraging machine learning models may improve the recognition of LPA^75^, ^76^ and hence provide a clearer picture of its impact on outcomes. Additionally, analyzing accelerometer data using a continuous intensity spectrum with higher resolution bands may provide new knowledge about the association between physical activity and health in children and adolescents.^77^

Most of the existing research into the relationship between 24‐hour movement behaviors and adiposity is based on the association with BMI *z*‐scores. Although BMI may provide a reasonable proxy for adiposity in children, its validity is dependent on several factors including ethnicity, body composition, and sex.^78^ Body mass index has a strong correlation with body fat in heavy children (>85th percentile for BMI); however, is less strongly correlated with BMI in children with a BMI < 85th percentile, where the association between fat‐free mass and BMI is stronger, which may mean that BMI can be inflated due to higher levels of fat‐free mass.^79^ Additionally, BMI is strongly associated with both fat‐mass and fat‐free mass in young children (i.e., 3–5 years),^80^ whereas BMI is more strongly associated with fat mass than fat‐free mass in older children and adolescents.^79^ Therefore, BMI may be an indicator of either higher fat mass or fat‐free mass in young children, which might explain why there was an inverse association between the amount of time spent in MVPA relative to other behaviors and BMI *z*‐score in children and adolescents, but not young children. The association between 24‐hour movement behaviors and BMI was only examined in young children in three studies and fat mass in one study, which found no significant associations.^66^ More research into the relationship between 24‐hour movement behaviors and adiposity in early childhood is needed to further clarify the relationship with 24‐hour movement in the early years. Among children and adolescents, the results from the meta‐analyses on percent body fat and fat‐mass index followed similar trends to BMI *z‐*score supporting the validity of the results observed for BMI *z*‐score.

The results of the current systematic review and meta‐analysis indicate important areas of focus for future research. First, there is an obvious need to examine the prospective association between 24‐hour movement behaviors and adiposity in children and adolescents. For example, future research may consider the longitudinal association between 24‐hour movement behaviors compositions and incidences of obesity in children and adolescents. Additionally, much of the extant research only examined BMI as an outcome. Additional assessments of adiposity including waist circumference and fat‐mass should be included in studies examining the association between 24‐hour movement behaviors and adiposity moving forward. This may be particularly important in the early years to tease out the counterintuitive results observed in the current meta‐analysis. Lastly, the current review examined all‐for‐one substitutions when examining the association between individual components of the 24‐hour movement behaviors and adiposity. A meta‐analysis of individual participant data to determine the association between one‐for‐one substitutions (i.e., compositional isotemporal substitution analysis) and adiposity indicators in children and adolescents could further demonstrate the optimal composition of 24‐hour movement behaviors to promote healthy adiposity outcomes.

This study provides the most comprehensive synthesis to date of the association between 24‐hour movement behavior composition with adiposity in children and adolescents. Nevertheless, there are several limitations that need to be considered. First, despite the growing application of compositional data analysis to the study of 24‐hour movement behaviors, only 16 studies met the inclusion criteria for the current review, and the number of studies included in each analysis ranged from three to 14. Additionally, only two studies reported on longitudinal associations, and the meta‐analyses were conducted on only cross‐sectional studies; therefore, it is not possible to draw conclusions about causality from the reported results. It is feasible, at least to some extent, that the results from the meta‐analysis could be a consequence of reverse causation. Additionally, several of the included studies used a convenience sampling method to recruit participants. Therefore, participants in many of the included studies may not be representative, and certain groups of children (such as those living in remote and rural areas) may have been actively excluded from included studies. Additionally, the majority of included studies examined adolescents and fewer studies examined the association between movement 24‐hour movement behaviors and indicators of adiposity in children and young children. Further, this study only included studies written in the English language. Excluding studies not written in the English language may have biased the results by excluding studies from countries where the English language is not widely spoken, which likely included countries where the population is not predominantly white. As a result of the above‐mentioned limitations in sampling, the generalizability of the results remains unclear. Another limitation is that none of the included studies assessed sedentary screen time, which is included in the 24‐Hour Movement Behavior Guidelines. Lastly, each of the studies applies various methodologies including using different brands of accelerometers and proprietary cut‐points to assess the intensity of waketime movement behaviors, assessing sleep duration using different wake–sleep algorithms, and including various covariates in statistical models. Additionally, only two studies controlled for the consumption of unhealthy foods, and no studies controlled for caloric intake. Although the studies are similar enough to warrant inclusion in a meta‐analysis, the differences in methodologies may contribute to between study heterogeneity among the included studies.

## 5 CONCLUSION

This systematic review and meta‐analysis is the first to quantitatively synthesize existing evidence examining the associations between 24‐hour movement behavior compositions and adiposity in children and youth. Results support the 24‐hour movement behavior guidelines by showing that getting more sleep, limiting sedentary time, and engaging in higher levels of MVPA are inversely associated with adiposity. However, most of the research is based on BMI z‐score. An interesting result of the current review is that spending more time in LPA at the expense of other behaviors may not be beneficial for adiposity outcomes. These results suggest that targeting behavior change to increase levels of MVPA specifically, rather than overall physical activity or LPA, while simultaneously preserving time for sleep and reducing sedentary time may be most effective in decreasing rates of overweight and obesity among children and adolescents. More research is needed examining direct indicators of adiposity, such as fat mass, to further elucidate the association between 24‐hour movement behaviors and adiposity in children and adolescents. Additionally, most of the research to date has been conducted on samples of adolescents. More research is also needed in younger children, where the association between different parts of 24‐hour movement behavior compositions and adiposity is less well established.

## CONFLICT OF INTEREST STATEMENT

The authors declare no conflicts of interest.

## Supporting information


**Appendix S1.** Supporting Information.

## Data Availability

The data necessary to complete this analysis are publicly available in the original publications included in the review, or from the authors of the original publications upon request. The code to process the data are available from the lead author upon request.
