# Supplementary material for: Association between 24‐hour movement behaviors and adiposity in children and adolescents: A compositional data meta‐analysis
Source: Obes Rev. 2025 Jan 20;26(5):e13884. doi: 10.1111/obr.13884 (PMC11964791; doi:10.1111/obr.13884)
Supplement: Supplementary file 1 — Appendix S1. Supporting Information. [file OBR-26-e13884-s001.pdf]

Appendix A – Search terms used for systematic review

24-hour OR movement behavio?r* OR physical behavio?r* OR time-use OR composition* analys* OR (physical activity AND sleep AND sedentary

AND

body mass OR bmi OR fat mass OR body weight OR waist circumference OR anthropometric OR body composition OR adipos* OR overweight OR obesity OR height-to-weight OR body fat

AND

child* OR preschool* OR adolescen* OR teen* OR youth* OR boy* OR girl* OR school-age* OR student* OR p?ediatric or early year* OR elementary school* OR primary school* OR secondary school* OR high school* OR young people OR young person OR toddler*

Appendix B – Studies excluded after full text review

Authors	Year	Title	DOI	Notes
Aadland, Eivind; Kvalheim, Olav Martin; Anderssen, Sigmund Alfred; Resaland, Geir Kare; Andersen, Lars Bo	2019	Multicollinear physical activity accelerometry data and associations to cardiometabolic health: challenges, pitfalls, and potential solutions	https://dx.doi.org/10.1186/s12966-019-0836-z	Exclusion reason: Did not assess all 24-hour movement behaviours
Alanazi, Yazeed A.; Sousa-Sa, Eduarda; Chong, Kar Hau; Parrish, Anne-Maree; Okely, Anthony D.	2021	Systematic Review of the Relationships between 24-Hour Movement Behaviours and Health Indicators in School-Aged Children from Arab-Speaking Countries	https://dx.doi.org/10.3390/ijerph18168640	Exclusion reason: Ineligible publication type
Alhowikan, Abdulrahman Mohammed; Alsharqawy, Nagwa Ebrahim; Alazmaa, Nouf Nawaf; Saeed, Abdullah Ibrahim; Alhazzani, Yasser Abdullah; Alhowaish, Nasser Yousef; Yahya, Abdulaziz Fahad Bin; Alhozaimi, Zeyad Ahmed; Aleid, Naif Eid; Alhussain, Maha H.	2023	Lifestyle habits and obesity indices among male adolescents in Riyadh, Saudi Arabia	https://dx.doi.org/10.1038/s41598-023-37920-5	Exclusion reason: Did not assess all 24-hour movement behaviours
Amigo, Isaac; Pe a, Elsa; Errasti, Jos Manuel; Busto, Raquel	2016	Sedentary versus active leisure activities and their relationship with sleeping habits and body mass index in children of 9 and 10 years of age	10.1177/1359105314556161	Exclusion reason: Did not assess all 24-hour movement behaviours
Aragon-Martin, Ruben; Gomez-Sanchez, Maria Del Mar; Martinez-Nieto, Jose Manuel; Novalbos-Ruiz, Jose Pedro; Segundo-Iglesias, Carmen; Santi-Cano, Maria Jose; Castro-Pinero, Jose; Lineros-Gonzalez, Carmen; Hernan-Garcia, Mariano; Schwarz-Rodriguez, Monica; Jimenez-Pavon, David; Rodriguez-Martin, Amelia	2022	Independent and Combined Association of Lifestyle Behaviours and Physical Fitness with Body Weight Status in Schoolchildren	https://dx.doi.org/10.3390/nu14061208	Exclusion reason: Did not assess all 24-hour movement behaviours
Babatunde, R. R.; Rege, S.; Chavan, S.	2023	TIME USE PATTERNS IN CHILDREN WITH AND WITHOUT CHILDHOOD OBESITY	10.22159/ajpcr.2023.v16i1.46344	Exclusion reason: Did not assess all 24-hour movement behaviours
Berglind, D.; Ljung, R.; Tynelius, P.; Brooke, H. L.	2018	Cross-sectional and prospective associations of meeting 24-h movement guidelines with overweight and obesity in preschool children	https://dx.doi.org/10.1111/ijpo.12265	Exclusion reason: Did not assess all 24-hour movement behaviours
Bianchim, M. S.; McNarry, M. A.; Holland, A.; Cox, N. S.; Dreger, J.; Barker, A. R.; Williams, C. A.; Denford, S.; Mackintosh, K. A.	2022	A Compositional Analysis of Physical Activity, Sedentary Time, and Sleep and Associated Health Outcomes in Children and Adults with Cystic Fibrosis	10.3390/ijerph19095155	Exclusion reason: Ineligible outcomes

Bird, Madeleine; Datta, Geetanjali D.; Chinerman, Deanna; Kakinami, Lisa; Mathieu, Marie-Eve; Henderson, Melanie; Barnett, Tracie A.	2022	Associations of neighborhood walkability with moderate to vigorous physical activity: an application of compositional data analysis comparing compositional and non-compositional approaches	https://dx.doi.org/10.1186/s12966-022-01256-6	Exclusion reason: Ineligible outcomes
Bourke, Matthew; Vanderloo, Leigh M.; Irwin, Jennifer D.; Burke, Shauna M.; Johnson, Andrew M.; Driediger, Molly; Timmons, Brian W.; Tucker, Patricia	2022	Association between childcare movement behaviour compositions with health and development among preschoolers: Finding the optimal combinations of physical activities and sedentary time	https://dx.doi.org/10.1080/02640414.2022.2134969	Exclusion reason: Did not assess all 24-hour movement behaviours
Burns, R. D.; Bai, Y.; Fu, Y.; Brusseau, T. A.	2020	Associations of adolescent lifestyle behaviors with body mass index within a nationally representative sample of US adolescents: a quantile regression analysis	https://dx.doi.org/10.1016/j.puhe.2019.10.002	Exclusion reason: Did not use compositional data analysis
Burns, Ryan D.; Brusseau, Timothy A.; You, Fu; Yang, Bai; Wonwoo, Byun	2021	Segmented School Physical Activity And Weight Status In Children: Application Of Compositional Data Analysis		Exclusion reason: Did not assess all 24-hour movement behaviours
Burns, Ryan Donald; Brusseau, Timothy A.; Bai, Yang; Byun, Wonwoo	2021	Segmented School Physical Activity and Weight Status in Children: Application of Compositional Data Analysis	https://dx.doi.org/10.3390/ijerph18063243	Exclusion reason: Did not assess all 24-hour movement behaviours
Burns, Ryan; Kim, Youngwon; Byun, Wonwoo; Brusseau, Timothy	2019	Associations of School Day Sedentary Behavior and Physical Activity With Gross Motor Skills: Use of Compositional Data Analysis	https://dx.doi.org/10.1123/jpah.2018-0549	Exclusion reason: Ineligible outcomes
Callender, Laura K.; Borghese, Michael M.; Janssen, Ian	2021	Which intensities, types, and patterns of movement behaviors are most strongly associated with cardiometabolic risk factors among children?	https://dx.doi.org/10.1016/j.jshs.2019.06.006	Exclusion reason: Did not use compositional data analysis
Carson, V.; Tremblay, M. S.; Chaput, J. P.; Chastin, S.	2016	Associations between physical activity, sedentary time, sleep, and adiposity indicators of Canadian children and youth using compositional analyses		Exclusion reason: Duplicate
Carson, Valerie; Faulkner, Guy; Sabiston, Catherine M.; Tremblay, Mark S.; Leatherdale, Scott T.	2015	Patterns of movement behaviors and their association with overweight and obesity in youth	https://dx.doi.org/10.1007/s00038-015-0685-8	Exclusion reason: Did not assess all 24-hour movement behaviours
Carson, Valerie; Tremblay, Mark S.; Chaput, Jean-Philippe; McGregor, Duncan; Chastin, Sebastien	2019	Compositional analyses of the associations between sedentary time, different intensities of physical activity, and cardiometabolic biomarkers among	https://dx.doi.org/10.1371/journal.pone.0220009	Exclusion reason: Did not assess all 24-hour movement behaviours

		children and youth from the United States		
Castro, Joao Antonio Chula; Nunes, Heloyse Elaine Gimenes; Silva, Diego Augusto Santos	2016	Prevalence of abdominal obesity in adolescents: association between sociodemographic factors and lifestyle	https://dx.doi.org/10.1016/j.rpped.2016.01.003	Exclusion reason: Did not assess all 24-hour movement behaviours
Chaput, J. P.; Barnes, J. D.; Tremblay, M. S.; Fogelholm, M.; Hu, G.; Lambert, E. V.; Maher, C.; Maia, J.; Olds, T.; Onywera, V.; Sarmiento, O. L.; Standage, M.; Tudor-Locke, C.; Katzmarzyk, P. T.	2018	Inequality in physical activity, sedentary behaviour, sleep duration and risk of obesity in children: a 12-country study	https://dx.doi.org/10.1002/osp4.271	Exclusion reason: Did not assess all 24-hour movement behaviours
Chaput, J. P.; Saunders, T. J.; Carson, V.	2017	Interactions between sleep, movement and other non-movement behaviours in the pathogenesis of childhood obesity	https://dx.doi.org/10.1111/obr.12508	Exclusion reason: Ineligible publication type
Chaput, Jean-Philippe; Colley, Rachel C.; Aubert, Salome; Carson, Valerie; Janssen, Ian; Roberts, Karen C.; Tremblay, Mark S.	2017	Proportion of preschool-aged children meeting the Canadian 24-Hour Movement Guidelines and associations with adiposity: results from the Canadian Health Measures Survey	https://dx.doi.org/10.1186/s12889-017-4854-y	Exclusion reason: Did not assess all 24-hour movement behaviours
Chong, Kar Hau; Parrish, Anne-Maree; Cliff, Dylan P.; Dumuid, Dorothea; Okely, Anthony D.	2022	Changes in 24-hour movement behaviours during the transition from primary to secondary school among Australian children		Exclusion reason: Ineligible outcomes
Clarke, Anna E.; Carson, Valerie; Chaput, Jean-Philippe; Colley, Rachel C.; Roberts, Karen C.; Rollo, Scott; Tremblay, Mark S.; Janssen, Ian	2021	Meeting Canadian 24-Hour Movement Guideline recommendations and risk of all-cause mortality		Exclusion reason: Ineligible population
Collings, Paul; Westgate, Kate; V ist , Juuso; Wijndaele, Katrien; Atkin, Andrew; Haapala, Eero; Lintu, Niina; Laitinen, Tomi; Ekelund, Ulf; Brage, Soren; Lakka, Timo	2017	Cross-Sectional Associations of Objectively-Measured Physical Activity and Sedentary Time with Body Composition and Cardiorespiratory Fitness in Mid-Childhood: The PANIC Study		Exclusion reason: Did not assess all 24-hour movement behaviours
da Costa, B. G. G.; Chaput, J. P.; Lopes, M. V. V.; Gaya, A. R.; Silva, D. A. S.; Silva, K. S.	2021	Association between sociodemographic, dietary, and substance use factors and accelerometer-measured 24-hour movement behaviours in Brazilian adolescents	10.1007/s00431-021-04112-0	Exclusion reason: Ineligible outcomes
De Souza, A. A.; Tassitano, R. M.; Mota, J. A.; Martins, C. D.	2021	Association between obesity indicators and clusters of physical activity and sedentary behavior	10.1159/000515911	Exclusion reason: Ineligible publication type
Del Pozo-Cruz, Borja; Gant, Nicholas; Del Pozo-Cruz, Jesus; Maddison, Ralph	2017	Relationships between sleep duration, physical activity and body mass index in	https://dx.doi.org/10.1371/journal.pone.0184472	Exclusion reason: Did not use compositional data analysis

		young New Zealanders: An isotemporal substitution analysis		
Ding, Caicui; Fan, Jing; Yuan, Fan; Feng, Ganyu; Gong, Weiyan; Song, Chao; Ma, Yanning; Chen, Zheng; Liu, Ailing	2022	Association between Physical Activity, Sedentary Behaviors, Sleep, Diet, and Adiposity among Children and Adolescents in China	https://dx.doi.org/10.1159/000519268	Exclusion reason: Did not assess all 24-hour movement behaviours
Dooley, E. E.; Gabriel, K. P.; Kohl, H. W.; Durand, C. P.; Hoelscher, D. M.; Byrd-Williams, C. E.	2020	Adiposity, cardiovascular, and health-related quality of life indicators and the reallocation of waking movement behaviors in preschool children with overweight and obesity: An isotemporal data analysis	10.1371/journal.pone.0242088	Exclusion reason: Did not use compositional data analysis
Draper, C. E.; Tomaz, S. A.; Cook, C. J.; Jugdav, S. S.; Ramsammy, C.; Besharati, S.; Heerden, A.; Vilakazi, K.; Cockcroft, K.; Howard, S. J.; Okely, A. D.	2020	Understanding the influence of 24-hour movement behaviours on the health and development of preschool children from low-income South African settings: The SUNRISE pilot study	10.17159/2078-516X/2020/v32i1a8415	Exclusion reason: Did not use compositional data analysis
D'Souza, Ninoshka J.; Downing, Katherine; Zheng, Miaobing; Abbott, Gavin; Lioret, Sandrine; Campbell, Karen J.; Hesketh, Kylie D.	2023	Cross-sectional and prospective associations between behavioural patterns and adiposity in school-aged children	https://dx.doi.org/10.1017/S136898002300112X	Exclusion reason: Did not use compositional data analysis
Dumuid, D.; Mart n-Fern ndez, J. A.; Ellul, S.; Kenett, R. S.; Wake, M.; Simm, P.; Baur, L.; Olds, T.	2021	Analysing body composition as compositional data: An exploration of the relationship between body composition, body mass and bone strength	10.1177/0962280220955221	Exclusion reason: Did not assess all 24-hour movement behaviours
Dumuid, Dorothea; Maher, Carol; Lewis, Lucy K.; Stanford, Tyman E.; Martin Fernandez, Josep Antoni; Ratcliffe, Julie; Katzmarzyk, Peter T.; Barreira, Tiago V.; Chaput, Jean-Philippe; Fogelholm, Mikael; Hu, Gang; Maia, Jose; Sarmiento, Olga L.; Standage, Martyn; Tremblay, Mark S.; Tudor-Locke, Catrine; Olds, Timothy	2018	Human development index, children's health-related quality of life and movement behaviors: a compositional data analysis	https://dx.doi.org/10.1007/s11136-018-1791-x	Exclusion reason: Ineligible outcomes
Dumuid, Dorothea; Olds, Timothy; Lange, Katherine; Edwards, Ben; Lycett, Kate; Burgner, David P.; Simm, Peter; Dwyer, Terence; Le, Ha; Wake, Melissa	2022	Goldilocks Days: optimising children's time use for health and well-being	https://dx.doi.org/10.1136/jech-2021-216686	Exclusion reason: Duplicate
Dumuid, Dorothea; Olds, Timothy; Wake, Melissa; Lund Rasmussen, Charlotte; Pedisic, Zeljko; Hughes, Jim H.; Foster, David, Jr.; Walmsley, Rosemary; Atkin, Andrew J.; Straker, Leon; Fraysse, Francois;	2022	Your best day: An interactive app to translate how time reallocations within a 24-hour day are associated with health measures	https://dx.doi.org/10.1371/journal.pone.0272343	Exclusion reason: Ineligible outcomes

Smith, Ross T.; Neumann, Frank; Kenett, Ron S.; Jarle Mork, Paul; Bennett, Derrick; Doherty, Aiden; Stanford, Ty				
Dumuid, Dorothea; Pedisic, Zeljko; Palarea-Albaladejo, Javier; Martin-Fernandez, Josep Antoni; Hron, Karel; Olds, Timothy	2020	Compositional Data Analysis in Time-Use Epidemiology: What, Why, How	https://dx.doi.org/10.3390/ijerph17072220	Exclusion reason: Did not assess all 24-hour movement behaviours
Dumuid, Dorothea; Pedisic, Zeljko; Stanford, Tyman Everleigh; Martin-Fernandez, Josep-Antoni; Hron, Karel; Maher, Carol A.; Lewis, Lucy K.; Olds, Timothy	2019	The compositional isotemporal substitution model: A method for estimating changes in a health outcome for reallocation of time between sleep, physical activity and sedentary behaviour	https://dx.doi.org/10.1177/0962280217737805	Exclusion reason: Duplicate
Dumuid, Dorothea; Stanford, Tyman E.; Pedisic, Zeljko; Maher, Carol; Lewis, Lucy K.; Martin-Fernandez, Josep-Antoni; Katzmarzyk, Peter T.; Chaput, Jean-Philippe; Fogelholm, Mikael; Standage, Martyn; Tremblay, Mark S.; Olds, Timothy	2018	Adiposity and the isotemporal substitution of physical activity, sedentary time and sleep among school-aged children: a compositional data analysis approach	https://dx.doi.org/10.1186/s12889-018-5207-1	Exclusion reason: Duplicate
Dumuid, Dorothea; Wake, Melissa; Burgner, David; Tremblay, Mark S.; Okely, Anthony D.; Edwards, Ben; Dwyer, Terence; Olds, Timothy	2021	Balancing time use for children's fitness and adiposity: Evidence to inform 24-hour guidelines for sleep, sedentary time and physical activity	https://dx.doi.org/10.1371/journal.pone.0245501	Exclusion reason: Duplicate
Dumuid, Dorothea; Wake, Melissa; Clifford, Susan; Burgner, David; Carlin, John B.; Mensah, Fiona K.; Fraysse, Francois; Lycett, Kate; Baur, Louise; Olds, Timothy	2019	The Association of the Body Composition of Children with 24-Hour Activity Composition	https://dx.doi.org/10.1016/j.jpeds.2018.12.030	Exclusion reason: Duplicate
Dygryn, Jan; Medrano, Maria; Molina-Garcia, Pablo; Rubin, Lukas; Jakubec, Lukas; Janda, David; Gaba, Ales	2021	Associations of novel 24-h accelerometer-derived metrics with adiposity in children and adolescents	https://dx.doi.org/10.1186/s12199-021-00987-5	Exclusion reason: Did not assess all 24-hour movement behaviours
Estevan, Isaac; Clark, Cain; Molina-Garcia, Javier; Menescardi, Cristina; Barton, Victoria; Queralt, Ana	2022	Longitudinal association of movement behaviour and motor competence in childhood: A structural equation model, compositional, and isotemporal substitution analysis	https://dx.doi.org/10.1016/j.jsams.2022.05.010	Exclusion reason: Ineligible outcomes
Fairclough, Stuart J.; Dumuid, Dorothea; Mackintosh, Kelly A.; Stone, Genevieve; Dagger, Rebecca; Stratton, Gareth; Davies, Ian; Boddy, Lynne M.	2018	Adiposity, fitness, health-related quality of life and the reallocation of time between children's school day activity behaviours: A compositional data analysis	https://dx.doi.org/10.1016/j.pmedr.2018.07.011	Exclusion reason: Did not assess all 24-hour movement behaviours
Fairclough, Stuart J.; Hurter, Liezel; Dumuid, Dorothea; Gaba, Ales; Rowlands, Alex V.; Cruz, Borja Del Pozo; Cox, Ashley; Crotti, Matteo; Foweather, Lawrence; Graves, Lee E. F.; Jones, Owen;	2022	The Physical Behaviour Intensity Spectrum and Body Mass Index in School-Aged Youth: A Compositional Analysis of Pooled Individual Participant Data	https://dx.doi.org/10.3390/ijerph19148778	Exclusion reason: Did not assess all 24-hour movement behaviours

McCann, Deborah A.; Noonan, Robert J.; Owen, Michael B.; Rudd, James R.; Taylor, Sarah L.; Tyler, Richard; Boddy, Lynne M.				
Felzer-Kim, Isabella T.; Hauck, Janet L.	2020	Sleep duration associates with moderate-to-vigorous intensity physical activity and body fat in 1- to 3-year-old children	https://dx.doi.org/10.1016/j.infbeh.2019.101392	Exclusion reason: Did not use compositional data analysis
Gaba, Ales; Dygryn, Jan; Stefelova, Nikola; Rubin, Lukas; Hron, Karel; Jakubec, Lukas	2021	Replacing school and out-of-school sedentary behaviors with physical activity and its associations with adiposity in children and adolescents: a compositional isotemporal substitution analysis	https://dx.doi.org/10.1186/s12199-021-00932-6	Exclusion reason: Duplicate
Gaba, Ales; Pedisic, Zeljko; Stefelova, Nikola; Dygryn, Jan; Hron, Karel; Dumuid, Dorothea; Tremblay, Mark	2020	Sedentary behavior patterns and adiposity in children: a study based on compositional data analysis	https://dx.doi.org/10.1186/s12887-020-02036-6	Exclusion reason: Did not assess all 24-hour movement behaviours
Godoy-Cumillaf, Andres; Fuentes-Merino, Paola; Farias-Valenzuela, Claudio; Duclos-Bastias, Daniel; Giakoni-Ramirez, Frano; Bruneau-Chavez, Jose; Merellano-Navarro, Eugenio	2023	The Association between Sedentary Behavior, Physical Activity, and Physical Fitness with Body Mass Index and Sleep Time in Chilean Girls and Boys: A Cross-Sectional Study	https://dx.doi.org/10.3390/children10060981	Exclusion reason: Did not use compositional data analysis
Grgic, Jozo; Dumuid, Dorothea; Bengoechea, Enrique Garcia; Shrestha, Nipun; Bauman, Adrian; Olds, Timothy; Pedisic, Zeljko	2018	Health outcomes associated with reallocations of time between sleep, sedentary behaviour, and physical activity: a systematic scoping review of isotemporal substitution studies	https://dx.doi.org/10.1186/s12966-018-0691-3	Exclusion reason: Ineligible publication type
Haszard, J.; Morrison, S.; Jackson, R.; Meredith-Jones, K.; Galland, B.; Taylor, R.	2022	Estimating the influence of sleep loss on dietary intake in children: A longitudinal compositional analysis using data from a randomized crossover trial	10.1111/obr.13502	Exclusion reason: Ineligible publication type
Hinkley, Trina; Timperio, Anna; Watson, Amanda; Duckham, Rachel L.; Okely, Anthony D.; Cliff, Dylan; Carver, Alison; Hesketh, Kylie D.	2020	Prospective associations with physiological, psychosocial and educational outcomes of meeting Australian 24-Hour Movement Guidelines for the Early Years	https://dx.doi.org/10.1186/s12966-020-00935-6	Exclusion reason: Did not assess all 24-hour movement behaviours
Hossain, Mohammad Sorowar; Raheem, Enayetur; Okely, Anthony D.	2023	24-hour movement guidelines and weight status among preschool-aged children in Bangladesh: A community-level cross-sectional study	https://dx.doi.org/10.1002/brb3.3094	Exclusion reason: Did not assess all 24-hour movement behaviours
Huang, S.; Huang, Y.; Gu, Y.; Chen, H.; Lv, R.; Wu, S.; Song, P.; Hu, L.; Yuan, C.	2022	Association of Adherence to 24-Hour Movement Guidelines With Childhood Overweight and Obesity	10.1002/oby.23626	Exclusion reason: Ineligible publication type

Huang, Siyi; Huang, Yuhui; Gu, Yuxuan; Chen, Hui; Lv, Rongxia; Wu, Shiyi; Song, Peige; Zhao, Dong; Hu, Liang; Yuan, Changzheng	2023	Adherence to 24-Hour Movement Guidelines in Relation to the Risk of Overweight and Obesity Among Children and Adolescents	https://dx.doi.org/10.1016/j.jadohealth.2023.06.009	Exclusion reason: Did not assess all 24-hour movement behaviours
Huang, Wendy Yajun; Wong, Stephen Heung-Sang; He, Gang; Salmon, J. O.	2016	Isotemporal Substitution Analysis for Sedentary Behavior and Body Mass Index		Exclusion reason: Did not use compositional data analysis
Hui, Stanley Sai-Chuen; Zhang, Ru; Suzuki, Koya; Naito, Hisashi; Balasekaran, Govindasamy; Song, Jong Kook; Park, Soo Yeon; Liou, Yiing Mei; Lu, Dajiang; Poh, Bee Koon; Kijboonchoo, Kallaya; Thasanasuwan, Wiyada	2021	The associations between meeting 24-hour movement guidelines and adiposity in Asian Adolescents: The Asia-Fit Study	https://dx.doi.org/10.1111/sms.13893	Exclusion reason: Did not assess all 24-hour movement behaviours
Jalali-Farahani, Sara; Amiri, Parisa; Chin, Yit Siew	2016	Are physical activity, sedentary behaviors and sleep duration associated with body mass index-for-age and health-related quality of life among high school boys and girls?	https://dx.doi.org/10.1186/s12955-016-0434-6	Exclusion reason: Did not assess all 24-hour movement behaviours
Jaskova, Paulina; Palarea-Albaladejo, Javier; Gaba, Ales; Dumuid, Dorothea; Pedisic, Zeljko; Pelclova, Jana; Hron, Karel	2023	Compositional functional regression and isotemporal substitution analysis: Methods and application in time-use epidemiology	https://dx.doi.org/10.1177/09622802231192949	Exclusion reason: Did not use compositional data analysis
Ji, Meimei; Tang, Amber; Zhang, Yefu; Zou, Jiaojiao; Zhou, Guangyu; Deng, Jing; Yang, Lina; Li, Mingzhi; Chen, Jihua; Qin, Hong; Lin, Qian	2018	The Relationship between Obesity, Sleep and Physical Activity in Chinese Preschool Children	https://dx.doi.org/10.3390/ijerph15030527	Exclusion reason: Did not use compositional data analysis
Julian, Valerie; Haschke, Ferdinand; Fearnbach, Nicole; Gomahr, Julian; Pixner, Thomas; Furthner, Dieter; Weghuber, Daniel; Thivel, David	2022	Effects of Movement Behaviors on Overall Health and Appetite Control: Current Evidence and Perspectives in Children and Adolescents	https://dx.doi.org/10.1007/s13679-021-00467-5	Exclusion reason: Ineligible publication type
Kariippanon, Katharina E.; Aguilar-Farias, Nicolas; El Hamdouchi, Asmaa; Hongyan, Guan; Lubree, Himangi; Okely, Anthony D.; Tremblay, Mark S.; Draper, Catherine E.	2023	The voices of children on movement behaviours: implications for promoting international guidelines to support obesity-prevention efforts	https://dx.doi.org/10.1016/S2214-109X(23)00100-6	Exclusion reason: Ineligible publication type
Katzmarzyk, P.; Staiano, A.	2017	Relationship between meeting 24-hour movement guidelines and measures of adiposity in children	10.1159/000468958	Exclusion reason: Ineligible publication type
Katzmarzyk, Peter T.; Staiano, Amanda E.	2017	Relationship Between Meeting 24-Hour Movement Guidelines and Cardiometabolic Risk Factors in Children	https://dx.doi.org/10.1123/jpah.2017-0090	Exclusion reason: Did not assess all 24-hour movement behaviours

Kemp, Byron J.; Dumuid, Dorothea; Chong, Kar Hau; Parrish, Anne-Maree; Cliff, Dylan	2023	Cross-sectional and longitudinal associations of domain-specific physical activity composition with health-related quality of life in childhood and adolescence in Australia	https://dx.doi.org/10.1186/s12966-023-01466-6	Exclusion reason: Did not assess all 24-hour movement behaviours
Khan, Asaduzzaman; Lee, Eun-Young; Tremblay, Mark S.	2021	Meeting 24-h movement guidelines and associations with health related quality of life of Australian adolescents		Exclusion reason: Did not assess all 24-hour movement behaviours
Kim, Hyunshik; Ma, Jiameng; Harada, Kenji; Lee, Sunkyoung; Gu, Ying	2020	Associations between Adherence to Combinations of 24-h Movement Guidelines and Overweight and Obesity in Japanese Preschool Children	https://dx.doi.org/10.3390/ijerph17249320	Exclusion reason: Did not assess all 24-hour movement behaviours
Koh, D.; Poh, B.; Abd Talib, R.; Zulhanif, M.; Hafizah Yatiman, N.; Summerbell, C.; Hillier Brown, F.; Gibson, E. L.	2019	Physical activity behaviour among preschool children: Preliminary baseline analysis from ToyBox Study Malaysia	10.1159/000489691	Exclusion reason: Ineligible publication type
Kracht, Chelsea L.; Katzmarzyk, Peter T.; Champagne, Catherine M.; Broyles, Stephanie T.; Hsia, Daniel S.; Newton Jr, Robert L.; Staiano, Amanda E.	2023	Association between Sleep, Sedentary Time, Physical Activity, and Adiposity in Adolescents: A Prospective Observational Study		Exclusion reason: Did not use compositional data analysis
Lee, Shoo Thien; Wong, Jyh Eiin; Chan, Geraldine K. L.; Poh, Bee Koon	2021	Association between Compliance with Movement Behavior Guidelines and Obesity among Malaysian Preschoolers	https://dx.doi.org/10.3390/ijerph18094611	Exclusion reason: Did not assess all 24-hour movement behaviours
Lemes, Vanilson Batista; Gaya, Adroaldo Cezar Araujo; Gaya, Anelise Reis	2023	Comparison of 24-h movement behavior, health-related quality of life, and waist to height ratio between adolescents with healthy body mass index (BMI) and adolescents with BMI in the cardiometabolic risk zone		Exclusion reason: Did not use compositional data analysis
Lemos, Luis; Clark, Cain; Brand, Caroline; Pessoa, Maria Luiza; Gaya, Anelise; Mota, Jorge; Duncan, Michael; Martins, Clarice	2021	24-hour movement behaviors and fitness in preschoolers: A compositional and isotemporal reallocation analysis	https://dx.doi.org/10.1111/sms.13938	Exclusion reason: Ineligible outcomes
Leppanen, Marja H.; Haapala, Eero A.; Vaisto, Juuso; Ekelund, Ulf; Brage, Soren; Kilpelainen, Tuomas O.; Lakka, Timo A.	2022	Longitudinal and cross-sectional associations of adherence to 24-hour movement guidelines with cardiometabolic risk	https://dx.doi.org/10.1111/sms.14081	Exclusion reason: Did not assess all 24-hour movement behaviours
Liberali, Rafaela; Del Castanhel, Flavia; Kupek, Emil; Assis, Maria Alice Altenburg de	2021	Latent Class Analysis of Lifestyle Risk Factors and Association with Overweight and/or Obesity in Children and Adolescents: Systematic Review	https://dx.doi.org/10.1089/chi.2020.0115	Exclusion reason: Ineligible publication type
Liu, Y.; Tan, S.; Zou, G.	2022	Correlation analysis between daily behavior and physical health of middle school students in Tianjin	10.16835/j.cnki.1000-9817.2022.10.027	Exclusion reason: Not written in English

Lopez-Gil, Jose Francisco; Tapia-Serrano, Miguel Angel; Sevil-Serrano, Javier; Sanchez-Miguel, Pedro Antonio; Garcia-Hermoso, Antonio	2023	Are 24-hour movement recommendations associated with obesity-related indicators in the young population? A meta-analysis	https://dx.doi.org/10.1002/oby.23848	Exclusion reason: Ineligible publication type
Lucas-de la Cruz, Lidia; Martinez-Vizcaino, Vicente; Canete Garcia-Prieto, Jorge; Arias-Palencia, Natalia; Diez-Fernandez, Ana; Milla-Tobarra, Marta; Notario-Pacheco, Blanca	2018	Movement behaviors and cardiometabolic risk in schoolchildren	https://dx.doi.org/10.1371/journal.pone.0207300	Exclusion reason: Did not use compositional data analysis
Manyanga, Taru; Barnes, Joel D.; Chaput, Jean-Philippe; Guerrero, Michelle; Katzmarzyk, Peter T.; Mire, Emily F.; Prista, Antonio; Tremblay, Mark S.; Iscole Research Group	2020	Body mass index and movement behaviors among schoolchildren from 13 countries across a continuum of human development indices: A multinational cross-sectional study	https://dx.doi.org/10.1002/ajhb.23341	Exclusion reason: Did not assess all 24-hour movement behaviours
Matricciani, Lisa; Dumuid, Dorothea; Paquet, Catherine; Fraysse, Francois; Wang, Yichao; Baur, Louise A.; Juonala, Markus; Ranganathan, Sarath; Lycett, Kate; Kerr, Jessica A.; Burgner, David; Wake, Melissa; Olds, Tim	2021	Sleep and cardiometabolic health in children and adults: examining sleep as a component of the 24-h day	https://dx.doi.org/10.1016/j.sleep.2020.12.001	Exclusion reason: Ineligible outcomes
McNeil, Jessica; Tremblay, Mark S.; Leduc, Genevieve; Boyer, Charles; Belanger, Priscilla; Leblanc, Allana G.; Borghese, Michael M.; Chaput, Jean-Philippe	2015	Objectively-measured sleep and its association with adiposity and physical activity in a sample of Canadian children	https://dx.doi.org/10.1111/jsr.12241	Exclusion reason: Did not assess all 24-hour movement behaviours
Micklesfield, Lisa K.; Hanson, Sara K.; Lobelo, Felipe; Cunningham, Solveig A.; Hartman, Terryl J.; Norris, Shane A.; Stein, Aryeh D.	2021	Adolescent physical activity, sedentary behavior and sleep in relation to body composition at age 18 years in urban South Africa, Birth-to-Twenty+ Cohort	https://dx.doi.org/10.1186/s12887-020-02451-9	Exclusion reason: Did not assess all 24-hour movement behaviours
Migueles, Jairo H.; Delisle Nystrom, Christine; Leppanen, Marja H.; Henriksson, Pontus; Lof, Marie	2022	Revisiting the cross-sectional and prospective association of physical activity with body composition and physical fitness in preschoolers: A compositional data approach	https://dx.doi.org/10.1111/ijpo.12909	Exclusion reason: Did not assess all 24-hour movement behaviours
Moitra, Panchali; Madan, Jagmeet; Verma, Preeti	2021	Independent and combined influences of physical activity, screen time, and sleep quality on adiposity indicators in Indian adolescents	https://dx.doi.org/10.1186/s12889-021-12183-9	Exclusion reason: Did not assess all 24-hour movement behaviours
Mota, Jessica Gomes; Clark, Cain Craig Truman; Bezerra, Thayna Alves; Lemos, Luis; Reuter, Cezane Priscilla; Mota, Jorge Augusto Pinto Silva; Duncan, Michael Joseph; Martins, Clarice Maria De Lucena	2020	Twenty-four-hour movement behaviours and fundamental movement skills in preschool children: A compositional and isotemporal substitution analysis	https://dx.doi.org/10.1080/02640414.2020.1770415	Exclusion reason: Ineligible outcomes

Munambah, N.; Gretschel, P.; Muchirahondo, F.; Chiwaridzo, M.; Chikwanha, T.; Kariippanon, K. E.; Chong, K. H.; Cross, P. L.; Draper, C. E.; Okely, A. D.	2021	24 hour movement behaviours and the health and development of pre-school children from Zimbabwean settings: the SUNRISE pilot study	https://dx.doi.org/10.17159/2078-516X/2021/v33i1a10864	Exclusion reason: Did not use compositional data analysis
Na, X. N.; Zhu, Z.; Chen, Y. Y.; Wang, D. P.; Wang, H. J.; Song, Y.; Ma, X. C.; Wang, P. Y.; Liu, A. P.	2020	[Associations of distribution of time spent in physical activity and sedentary behavior with obesity]	https://dx.doi.org/10.19723/j.issn.1671-167X.2020.03.014	Exclusion reason: Not written in English
Nagata, Jason M.; Smith, Natalia; Alsamman, Sana; Lee, Christopher M.; Dooley, Erin E.; Kiss, Orsolya; Ganson, Kyle T.; Wing, David; Baker, Fiona C.; Gabriel, Kelley Pettee	2023	Association of Physical Activity and Screen Time With Body Mass Index Among US Adolescents	https://dx.doi.org/10.1001/jamanetworkopen.2022.55466	Exclusion reason: Did not assess all 24-hour movement behaviours
O'Connor, S.; Dooley, E.; Shams-White, M.; Bowles, H.; Nebeling, L.; Saint-Maurice, P.	2022	24hr Time Use Behavior and Body Mass Index Among Adolescents: A Compositional Data Analysis Approach	10.1002/oby.23626	Exclusion reason: Ineligible publication type
Okely, Anthony D.; Ghersi, Davina; Loughran, Sarah P.; Cliff, Dylan P.; Shilton, Trevor; Jones, Rachel A.; Stanley, Rebecca M.; Sherring, Julie; Toms, Natalie; Eckermann, Simon; Olds, Timothy S.; Zhang, Zhiguang; Parrish, Anne-Maree; Kervin, Lisa; Downie, Sandra; Salmon, Jo; Bannerman, Clair; Needham, Tamie; Marshall, Elaine; Kaufman, Jordy; Brown, Layne; Wille, Janecke; Wood, Greg; Lubans, David R.; Biddle, Stuart J. H.; Pill, Shane; Hargreaves, Anthea; Jonas, Natalie; Schranz, Natasha; Campbell, Perry; Ingram, Karen; Dean, Hayley; Verrender, Adam; Ellis, Yvonne; Chong, Kar Hau; Dumuid, Dorothea; Katzmarzyk, Peter T.; Draper, Catherine E.; Lewthwaite, Hayley; Tremblay, Mark S.	2022	A collaborative approach to adopting/adapting guidelines The Australian 24-hour movement guidelines for children (5-12 years) and young people (13-17 years): An integration of physical activity, sedentary behaviour, and sleep	10.1186/s12966-021-01236-2	Exclusion reason: Ineligible publication type
Okely, Anthony D.; Tremblay, Mark S.; Reilly, John J.; Draper, Catherine E.; Bull, Fiona	2018	Physical activity, sedentary behaviour, and sleep: movement behaviours in early life	https://dx.doi.org/10.1016/S2352-4642(18)30070-1	Exclusion reason: Ineligible publication type
Pereira, Sara; Katzmarzyk, Peter T.; Gomes, Thayse Natacha; Borges, Alessandra; Santos, Daniel; Souza, Michele; dos Santos, Fernanda K.; Chaves, Raquel N.; Champagne, Catherine M.; Barreira, Tiago V.; Maia, Jose A. R.	2015	Profiling physical activity, diet, screen and sleep habits in Portuguese children	https://dx.doi.org/10.3390/nu7064345	Exclusion reason: Did not assess all 24-hour movement behaviours

Q. I. U. Yanping; Wang Lijuan; Zhou Yulan; Chen Huan; Liang Guo	2023	The association between 24h movement behaviors and fundamental motor skills of children based on compositional data analyses		Exclusion reason: Not written in English
Ramirez-Marrero, Farah A.; Hernandez-Torres, Emmanuel; Torres-Villela, Luis; Estrada-Oliver, Luis G.; Melendez-Nieves, Anthony	2022	Accelerometer-Based Physical Activity, Sedentary Time, and BMI among Preschoolers in Puerto Rico		Exclusion reason: Did not assess all 24-hour movement behaviours
Reis, L. N.; Reuter, C. P.; Brand, C.; Martin, C.; Fochesatto, C. F.; Borfe, L.; Mota, J.; Gaya, A.; Gaya, A. R.	2021	24-hour movement behaviors and cardiometabolic risk factors in children: A isotemporal substitution analysis	10.1159/000515911	Exclusion reason: Ineligible publication type
Reis, Luiza N.; Reuter, Cezane P.; Okely, Anthony; Brand, Caroline; Fochesatto, Camila F.; Martins, Clarice M. L.; Mota, Jorge; Gaya, Adroaldo C. A.; Gaya, Anelise R.	2023	Replacing screen time, with physical activity and sleep time: influence on cardiovascular indicators and inflammatory markers in Brazilian children	https://dx.doi.org/10.1016/j.jped.2023.10.007	Exclusion reason: Did not assess all 24-hour movement behaviours
Riso, Eva-Maria; Kull, Merike; Mooses, Kerli; Jurimae, Jaak	2018	Physical activity, sedentary time and sleep duration: associations with body composition in 10-12-year-old Estonian schoolchildren	https://dx.doi.org/10.1186/s12889-018-5406-9	Exclusion reason: Did not use compositional data analysis
Robusto, Kristi Marie	2018	Childhood obesity prevention: Is it a hop, jump, and a skip away? An exploratory study of the behavioral ecological model and the influence of contingencies on child body composition		Exclusion reason: Did not use compositional data analysis
Rollo, Scott; Antsygina, Olga; Tremblay, Mark S.	2020	The whole day matters: Understanding 24-hour movement guideline adherence and relationships with health indicators across the lifespan	https://dx.doi.org/10.1016/j.jshs.2020.07.004	Exclusion reason: Ineligible publication type
Roman-Vinas, Blanca; Chaput, Jean-Philippe; Katzmarzyk, Peter T.; Fogelholm, Mikael; Lambert, Estelle V.; Maher, Carol; Maia, Jose; Olds, Timothy; Onywera, Vincent; Sarmiento, Olga L.; Standage, Martyn; Tudor-Locke, Catrine; Tremblay, Mark S.; Iscole Research Group	2016	Proportion of children meeting recommendations for 24-hour movement guidelines and associations with adiposity in a 12-country study		Exclusion reason: Did not assess all 24-hour movement behaviours
Roscoe, Clare M. P.; Duncan, Michael J.; Clark, Cain C. T.	2021	The 24-h Movement Compositions in Weekday, Weekend Day or Four-Day Periods Differentially Associate with Fundamental Movement Skills	https://dx.doi.org/10.3390/children8100828	Exclusion reason: Ineligible outcomes
Rubin, Lukas; Gaba, Ales; Pelclova, Jana; Stefelova, Nikola; Jakubec, Lukas; Dygryn, Jan; Hron, Karel	2022	Changes in sedentary behavior patterns during the transition from childhood to adolescence and their association with		Exclusion reason: Did not assess all 24-hour movement behaviours

		adiposity: a prospective study based on compositional data analysis		
Said, Mohamed Ahmed; Shaab Alibrahim, Mohammed	2022	Physical activity, sedentary behaviors, and breakfast eating as factors influencing BMI in Saudi students, aged 10 to 15 years	https://dx.doi.org/10.1080/07853890.2022.2077429	Exclusion reason: Did not assess all 24-hour movement behaviours
Sampasa-Kanyinga, Hugues; Colman, Ian; Hamilton, Hayley A.; Chaput, Jean-Philippe	2020	Outdoor physical activity, compliance with the physical activity, screen time, and sleep duration recommendations, and excess weight among adolescents	https://dx.doi.org/10.1002/osp4.389	Exclusion reason: Did not assess all 24-hour movement behaviours
Santos, Rute; Zhang, Zhiguang; Pereira, Joao R.; Sousa-Sa, Eduarda; Cliff, Dylan P.; Okely, Anthony D.	2017	Compliance with the Australian 24-hour movement guidelines for the early years: associations with weight status	https://dx.doi.org/10.1186/s12889-017-4857-8	Exclusion reason: Did not use compositional data analysis
Saunders, Travis John; Gray, Casey Ellen; Poitras, Veronica Joan; Chaput, Jean-Philippe; Janssen, Ian; Katzmarzyk, Peter T.; Olds, Timothy; Connor Gorber, Sarah; Kho, Michelle E.; Sampson, Margaret; Tremblay, Mark S.; Carson, Valerie	2016	Combinations of physical activity, sedentary behaviour and sleep: relationships with health indicators in school-aged children and youth	https://dx.doi.org/10.1139/apnm-2015-0626	Exclusion reason: Ineligible publication type
Schott, Whitney; Aurino, Elisabetta; Penny, Mary E.; Behrman, Jere R.	2020	Time use and sexual maturity-related indicators differentially predict youth body mass indices, Peruvian girls versus boys	https://dx.doi.org/10.1111/nyas.14292	Exclusion reason: Did not assess all 24-hour movement behaviours
Shakir, Rima N.; Coates, Alison M.; Olds, Timothy; Rowlands, Alex; Tsiros, Margarita D.	2018	Not all sedentary behaviour is equal: Children's adiposity and sedentary behaviour volumes, patterns and types	https://dx.doi.org/10.1016/j.orcp.2018.09.001	Exclusion reason: Did not use compositional data analysis
Shi, Yan; Huang, Wendy Yajun; Sit, Cindy Hui-Ping; Wong, Stephen Heung-Sang	2020	Compliance With 24-Hour Movement Guidelines in Hong Kong Adolescents: Associations With Weight Status	https://dx.doi.org/10.1123/jpah.2019-0230	Exclusion reason: Did not use compositional data analysis
Smith, Elizabeth; Fazeli, Fatima; Wilkinson, Kate; Clark, Cain C. T.	2021	Physical behaviors and fundamental movement skills in British and Iranian children: An isotemporal substitution analysis	https://dx.doi.org/10.1111/sms.13837	Exclusion reason: Ineligible outcomes
St Laurent, C. W.; Spencer, R.	2020	The association of habitual physical activity with 24-hour sleep outcomes in preschoolers	10.1093/sleep/zsaa056.220	Exclusion reason: Ineligible publication type
Stefelova, Nikola; Dygryn, Jan; Hron, Karel; Gaba, Ales; Rubin, Lukas; Palarea-Albaladejo, Javier	2018	Robust Compositional Analysis of Physical Activity and Sedentary Behaviour Data	https://dx.doi.org/10.3390/ijerph15102248	Exclusion reason: Did not assess all 24-hour movement behaviours
Stoner, L.; Harding, R.; Hszard, J.; Meredith-Jones, K.; Saedi, P.; Skidmore, P.	2019	Associations between lifestyle behaviors and body composition in 9-11 year old	10.1161/circ.139.suppl_1.P054	Exclusion reason: Ineligible publication type

		New Zealand children: The moderating effect of parental resilience		
Sun, Yi; Liu, Yuan; Yin, Xiaojian; Li, Ming; Zhang, Ting; Zhang, Feng; Guo, Yaru; Sun, Pengwei	2023	Proportion of Chinese Children and Adolescents Meeting 24-Hour Movement Guidelines and Associations with Overweight and Obesity	https://dx.doi.org/10.3390/ijerph20021408	Exclusion reason: Did not assess all 24-hour movement behaviours
Swindell, Nils; Rees, Paul; Fogelholm, Mikael; Drummen, Mathijs; MacDonald, Ian; Martinez, J. Alfredo; Navas-Carretero, Santiago; Handjieva-Darlenska, Teodora; Boyadjieva, Nadka; Bogdanov, Georgi; Poppitt, Sally D.; Gant, Nicholas; Silvestre, Marta P.; Brand-Miller, Jennie; Schlicht, Wolfgang; Muirhead, Roslyn; Brodie, Shannon; Tikkanen, Heikki; Jalo, Elli; Westerterp-Plantenga, Margriet; Adam, Tanja; Vestentoft, Pia Siig; Larsen, Thomas M.; Raben, Anne; Stratton, Gareth	2020	Compositional analysis of the associations between 24-h movement behaviours and cardio-metabolic risk factors in overweight and obese adults with pre-diabetes from the PREVIEW study: Cross-sectional baseline analysis	10.1186/s12966-020-00936-5	Exclusion reason: Ineligible population
Tanaka, Chiaki; Tremblay, Mark S.; Okuda, Masayuki; Inoue, Shigeru; Tanaka, Shigeho	2020	Proportion of Japanese primary school children meeting recommendations for 24-h movement guidelines and associations with weight status	https://dx.doi.org/10.1016/j.orcp.2020.05.003	Exclusion reason: Did not assess all 24-hour movement behaviours
Tandon, Pooja S.; Sasser, Tyler; Gonzalez, Erin S.; Whitlock, Kathryn B.; Christakis, Dimitri A.; Stein, Mark A.	2019	Physical Activity, Screen Time, and Sleep in Children With ADHD	https://dx.doi.org/10.1123/jpah.2018-0215	Exclusion reason: Did not assess all 24-hour movement behaviours
Taylor, R. W.; Haszard, J. J.; Farmer, V. L.; Richards, R.; Te Morenga, L.; Meredith-Jones, K.; Mann, J. I.	2020	Do differences in compositional time use explain ethnic variation in the prevalence of obesity in children? Analyses using 24-hour accelerometry	https://dx.doi.org/10.1038/s41366-019-0377-1	Exclusion reason: Duplicate
tefelov , N.; Palarea-Albaladejo, J.; Hron, K.; G ba, A.; Dygr n, J.	2023	Compositional PLS biplot based on pivoting balances: an application to explore the association between 24-h movement behaviours and adiposity	10.1007/s00180-023-01324-w	Exclusion reason: Duplicate
Tomaz, Simone A.; Prioreschi, Alessandra; Watson, Estelle D.; McVeigh, Joanne A.; Rae, Dale E.; Jones, Rachel A.; Draper, Catherine E.	2019	Body Mass Index, Physical Activity, Sedentary Behavior, Sleep, and Gross Motor Skill Proficiency in Preschool Children From a Low- to Middle-Income Urban Setting	https://dx.doi.org/10.1123/jpah.2018-0133	Exclusion reason: Did not use compositional data analysis
Tremblay, Mark S.; Carson, Valerie; Chaput, Jean-Philippe; Connor Gorber, Sarah; Thy, Dinh; Duggan, Mary; Faulkner, Guy; Gray, Casey E.; Gruber, Reut; Janson, Katherine; Janssen, Ian; Katzmarzyk, Peter	2016	Canadian 24-Hour Movement Guidelines for Children and Youth: An Integration of Physical Activity, Sedentary Behaviour, and Sleep		Exclusion reason: Ineligible publication type

T.; Kho, Michelle E.; Latimer-Cheung, Amy E.; LeBlanc, Claire; Okely, Anthony D.; Olds, Timothy; Pate, Russell R.; Phillips, Andrea; Poitras, Veronica J.				
Tremblay, Mark S.; Chaput, Jean-Philippe; Adamo, Kristi B.; Aubert, Salome; Barnes, Joel D.; Choquette, Louise; Duggan, Mary; Faulkner, Guy; Goldfield, Gary S.; Gray, Casey E.; Gruber, Reut; Janson, Katherine; Janssen, Ian; Janssen, Xanne; Jaramillo Garcia, Alejandra; Kuzik, Nicholas; LeBlanc, Claire; MacLean, Joanna; Okely, Anthony D.; Poitras, Veronica J.; Rayner, Mary-Ellen; Reilly, John J.; Sampson, Margaret; Spence, John C.; Timmons, Brian W.; Carson, Valerie	2017	Canadian 24-Hour Movement Guidelines for the Early Years (0-4 years): An Integration of Physical Activity, Sedentary Behaviour, and Sleep	https://dx.doi.org/10.1186/s12889-017-4859-6	Exclusion reason: Ineligible publication type
Tye, Lauren S.; Scott, Tessa; Haszard, Jillian J.; Peddie, Meredith C.	2020	Physical Activity, Sedentary Behaviour and Sleep, and Their Association with BMI in a Sample of Adolescent Females in New Zealand	https://dx.doi.org/10.3390/ijerph17176346	Exclusion reason: Did not use compositional data analysis
Vale, S.; Rego, C.; Mota, J.	2018	Adherence 24hr movement guidelines and adiposity among portuguese preschoolers	10.1159/000494676	Exclusion reason: Ineligible publication type
Verswijveren, Simone J. J. M.; Lamb, Karen E.; Martin-Fernandez, Josep A.; Winkler, Elisabeth; Leech, Rebecca M.; Timperio, Anna; Salmon, Jo; Daly, Robin M.; Cerin, Ester; Dunstan, David W.; Telford, Rohan M.; Telford, Richard D.; Olive, Lisa S.; Ridgers, Nicola D.	2022	Using compositional data analysis to explore accumulation of sedentary behavior, physical activity and youth health	https://dx.doi.org/10.1016/j.jshs.2021.03.004	Exclusion reason: Did not assess all 24-hour movement behaviours
Wang, J.; Gong, T.	2021	Association between physical activity and sedentary behavior with the risk of overweight and obesity in primary school students	10.16835/j.cnki.1000-9817.2021.11.019	Exclusion reason: Not written in English
Wang, R.; Wu, B.; Gao, Y.; Li, H.; Zhang, T.	2023	Relationship between 24?hour movement behavior and obesity indicators in children and adolescents	10.16835/j.cnki.1000-9817.2023.01.006	Exclusion reason: Not written in English
Wang, Y.; Zhu, R.; Wang, Y.; Long, J.; Zhang, Y.	2021	Levels of 24-hour movement and associations with childhood obesity in Chinese school students	10.16835/j.cnki.1000-9817.2021.04.029	Exclusion reason: Not written in English
Wilhite, Katrina; Booker, Bridget; Huang, Bo-Huei; Antczak, Devan; Corbett, Lucy; Parker, Philip; Noetel, Michael; Rissel,	2023	Combinations of Physical Activity, Sedentary Behavior, and Sleep Duration and Their Associations With Physical,	https://dx.doi.org/10.1093/aje/kwac212	Exclusion reason: Ineligible publication type

Chris; Lonsdale, Chris; Del Pozo Cruz, Borja; Sanders, Taren		Psychological, and Educational Outcomes in Children and Adolescents: A Systematic Review		
Wilkie, Hannah J.; Standage, Martyn; Gillison, Fiona B.; Cumming, Sean P.; Katzmarzyk, Peter T.	2016	Multiple lifestyle behaviours and overweight and obesity among children aged 9-11 years: results from the UK site of the International Study of Childhood Obesity, Lifestyle and the Environment	https://dx.doi.org/10.1136/bmjopen-2015-010677	Exclusion reason: Did not assess all 24-hour movement behaviours
Wong, J. E.; Lee, S. T.; Koh, D.; Poh, B. K.	2022	Twenty-four-hour movement behaviour profiles: Comparison by obesity status among Malaysian schoolchildren	10.1111/obr.13503	Exclusion reason: Ineligible publication type
Wyszynska, Justyna; Matlosz, Piotr; Asif, Muhammad; Szybisty, Agnieszka; Lenik, Pawel; Deren, Katarzyna; Mazur, Artur; Herbert, Jaroslaw	2021	Association between objectively measured body composition, sleep parameters and physical activity in preschool children: a cross-sectional study	https://dx.doi.org/10.1136/bmjopen-2020-042669	Exclusion reason: Did not assess all 24-hour movement behaviours
Wyszynska, Justyna; Matlosz, Piotr; Szybisty, Agnieszka; Deren, Katarzyna; Mazur, Artur; Herbert, Jaroslaw	2021	The association of actigraphic sleep measures and physical activity with excess weight and adiposity in kindergarteners	https://dx.doi.org/10.1038/s41598-021-82101-x	Exclusion reason: Did not use compositional data analysis
Yang, Yide; Yuan, Shuqian; Liu, Qiao; Li, Feifei; Dong, Yanhui; Dong, Bin; Zou, Zhiyong; Ma, Jun; Baker, Julien S.; Li, Xianxiong; Liang, Wei	2022	Meeting 24-Hour Movement and Dietary Guidelines: Prevalence, Correlates and Association with Weight Status among Children and Adolescents: A National Cross-Sectional Study in China	https://dx.doi.org/10.3390/nu14142822	Exclusion reason: Did not assess all 24-hour movement behaviours
Zhang, T.; Li, H.; Zhang, Z.; Gao, Y.	2023	A systematic review of the association between 24-hour movement behavior and obesity in children and adolescents	10.16835/j.cnki.1000-9817.2023.01.005	Exclusion reason: Not written in English
Zhang, X. R.; Chen, T. J.; Ma, J.	2021	Associations of sleep duration with overweight and obesity in school-age children	10.11852/zgetbjzz2020-1604	Exclusion reason: Not written in English
Zhang, Yunting; Zhang, Donglan; Li, Xinyue; Ip, Patrick; Ho, Frederick; Jiang, Yanrui; Sun, Wanqi; Zhu, Qi; Zhu, Weiming; Zhang, Jun; Zhao, Hongyu; Wang, Guanghai; Shen, Xiaoming; Jiang, Fan	2017	Daily Time-Use Patterns and Obesity and Mental Health among Primary School Students in Shanghai: A Population-Based Cross-Sectional Study	https://dx.doi.org/10.1038/s41598-017-15102-4	Exclusion reason: Did not assess all 24-hour movement behaviours
Zhou, Lin; Liang, Wei; He, Yuxiu; Duan, Yanping; Rhodes, Ryan E.; Liu, Hao; Liang, Hongmei; Shi, Xiaowei; Zhang, Jun; Cheng, Yingzhe	2022	Relationship of 24-Hour Movement Behaviors with Weight Status and Body Composition in Chinese Primary School Children: A Cross-Sectional Study	https://dx.doi.org/10.3390/ijerph19148586	Exclusion reason: Did not assess all 24-hour movement behaviours

Zhu, Xihe; Healy, Sean; Haegele, Justin A.; Patterson, Freda	2020	Twenty-Four-Hour Movement Guidelines and Body Weight in Youth	https://dx.doi.org/10.1016/j.jpeds.2019.11.031	Exclusion reason: Did not assess all 24-hour movement behaviours

Appendix C – Study funding details and conflicts of interest

Study	Funding	Conflict of interest
Carson et al. (2016)[1]	Canadian Society for Exercise Physiology, Conference Board of Canada, Healthy Active Living and Obesity Research Group at the Children's Hospital of Eastern Ontario Research Institute, and the Public Health Agency of Canada. The research was supported by funds to the Canadian Research Data Centre Network (CRDCN) from the Social Sciences and Humanities Research Council (SSHRC), the Canadian Institutes of Health Research (CIHR), the Canadian Foundation for Innovation (CFI), and Statistics Canada.	No conflicts of interest declared
Carson et al. (2017)[2]	Canadian Research Data Centre Network (CRDCN) from the Social Sciences and Humanities Research Council (SSHRC), the Canadian Institutes of Health Research (CIHR), the Canadian Foundation for Innovation (CFI), and Statistics Canada.	No conflicts of interest declared
Chen et al. (2023)[3]	National Social Science Fund of China (No. 22TYB00517) and Shanghai Sport Science and Technology Project of Shanghai Administration of Sports (No. 22Q005).	No conflicts of interest declared
Domingues et al. (2022)[4]	Coordenação de Aperfeiçoamento de Pessoal de Nível Superior - Brasil (CAPES) - Finance Code 001	No conflicts of interest declared
Dumuid et al. (2018)[5]	The example dataset used in this study is from the International Study of Childhood Obesity, Lifestyle and Environment (ISCOLE), which was funded by The Coca-Cola Company.	No conflicts of interest declared
Fairclough et al. (2017)[6]	West Lancashire School Sport Partnership, West Lancashire Community Leisure Trust, and Edge Hill University.	No conflicts of interest declared
Haszard et al. (2020)[7]	None reported	No conflicts of interest declared
Healy et al., (2020)[8]	Institutional Development Award (IDeA) Center ofBiomedical Research Excellence from the National Insti-tute of General Medical Sciences of the National Insti-tutes of Health under grant number P20GM113125	No conflicts of interest declared
Kuzik et al. (2020)[9]	No funding received.	No conflicts of interest declared
McGee et al., (2020)[10]	Hospital for Sick Children, Grant/Award Number: Restracomp; Canadian Institutes of Health Research, Institute of Nutrition, Metabolism and Diabetes, Grant/Award Number: FHG 129919	No conflicts of interest declared
Migueles et al., (2023)[11]	Swedish Research Council (project no. 2012–2883, Marie Löf), the Swedish Research Council for Health, Working Life and Welfare (2012–0906, Marie Löf; 2021–00036, Jairo H Migueles), Bo and Vera Axson Johnsons Foundation and Karolinska Institutet (Marie Löf, Jairo H Migueles), and the Joanna Cocozza Foundation (Marie Löf).	No conflicts of interest declared
Ng. et al., (2021)[12]	National Health and Medical Research Council (project grants 1041352 and 1109355), The Royal Children's Hospital Foundation (2014-241), the Murdoch Children's Research Institute, The University of Melbourne, the National	No conflicts of interest declared

	Heart Foundation of Australia (100660), and the Financial Markets Foundation for Children (2014-055 and 2016-310).	
Rasmussen et al., (2023)[13]	Czech Science Foundation (18-09188S and 22-02392S).	No conflicts of interest declared
Talarico et al., (2018)[14]	Heart and Stroke Foundation of Canada.	No conflicts of interest declared
Taylor et al. (2018)[15]	Health Research Council of New Zealand (08/374, 12/281, 12/310) and the Southern District Health Board.	No conflicts of interest declared
Zhang et al. (2022)[16]	Key Project for Education of the National Social Science Foundation of China (grant number ALA190015).	No conflicts of interest declared

Appendix D – Study quality assessment for included studies

**Study**	**1**	**2**	**3**	**4**	**5**	**6**	**7**	**8**	**9**	**10**	**11**	**12**	**13**	**14**	**15**	**16**	**17**	**18**	**19**	**20**	**OVR**
(Carson, Tremblay, Chaput, & Chastin, 2016)	Y	Y	N	Y	Y	Y	N	Y	Y	Y	Y	Y	Y	N	Y	Y	Y	Y	Y	Y	0.85
(Carson, Tremblay, & Chastin, 2017)	Y	Y	N	Y	Y	Y	Y	Y	Y	Y	Y	Y	Y	Y	Y	Y	Y	Y	Y	Y	0.95
(Chen, Wang, Xin, Liang, & Zhou, 2023)	Y	Y	Y	Y	Y	N	N	Y	Y	Y	Y	Y	Y	N	Y	N	Y	Y	Y	Y	0.80
(Domingues, Diniz da Silva, Faria, de Sá Souza, & dos Santos Amorim, 2022)	N	Y	N	Y	Y	Y	N	Y	Y	Y	N	N	N	N	N	Y	Y	N	Y	Y	0.55
(Dumuid et al., 2017)	Y	Y	N	Y	Y	Y	Y	Y	Y	Y	Y	Y	Y	Y	Y	N	Y	Y	Y	Y	0.90
(Fairclough et al., 2017)	Y	Y	N	Y	Y	N	N	Y	Y	Y	N	Y	N	N	Y	Y	Y	N	N	Y	0.60
(Haszard et al., 2020)	Y	Y	N	Y	N	N	N	Y	Y	Y	Y	Y	N	N	Y	Y	Y	Y	N	N	0.60
(Healy, Brewer, Garcia, Daly, & Patterson, 2021)	Y	Y	N	Y	Y	N	Y	Y	Y	Y	Y	Y	Y	Y	Y	N	Y	Y	Y	Y	0.85
(Kuzik, Naylor, Spence, & Carson, 2020)	Y	Y	Y	Y	Y	N	N	Y	Y	Y	Y	Y	N	N	N	N	Y	Y	Y	Y	0.70
(McGee et al., 2020)	Y	Y	N	Y	Y	Y	N	Y	Y	Y	Y	Y	N	N	Y	N	Y	Y	Y	Y	0.75
(Migueles et al., 2023)	Y	Y	N	Y	Y	Y	N	Y	Y	Y	N	Y	Y	N	Y	Y	Y	Y	Y	Y	0.80
(Ng et al., 2021)	Y	Y	N	Y	Y	Y	N	Y	Y	Y	Y	Y	N	Y	N	Y	Y	Y	Y	Y	0.80
(Lund Rasmussen et al., 2023)	Y	Y	N	Y	Y	N	N	Y	Y	Y	N	Y	N	N	Y	N	Y	Y	Y	Y	0.65
(Talarico & Janssen, 2018)	Y	Y	N	Y	Y	Y	N	Y	Y	Y	Y	Y	Y	N	Y	Y	Y	Y	Y	Y	0.85
(Taylor et al., 2018)	Y	Y	N	Y	Y	Y	Y	Y	Y	Y	Y	Y	Y	Y	Y	Y	Y	Y	Y	Y	0.95
(Zhang, Li, Li, Zhang, & Zhang, 2022)	Y	Y	Y	Y	Y	N	N	Y	Y	Y	Y	Y	Y	N	Y	N	Y	Y	Y	Y	0.80

Criteria: *1*. Were the aims/objectives of the study clear? *2*. Was the study design appropriate for the stated aim(s)? *3*. Was the sample size justified? *4*. Was the target/reference population clearly defined? (Is it clear who the research was about?) *5*. Was the sample frame taken from an appropriate population base so that it closely represented the target/reference population under investigation? *6.* Was the selection process likely to select subjects/participants that were representative of the target/reference population under investigation? *7*. Were measures undertaken to address and categorise non-responders? *8*. Were the risk factor and outcome variables measured appropriate to the aims of the study? *9*. Were the risk factor and outcome variables measured correctly using instruments/ measurements that had been trialled, piloted or published previously? *10.* Is it clear what was used to determined statistical significance and/or precision estimates? (eg, p values, CIs) *11*. Were the methods (including statistical methods) sufficiently described to enable them to be repeated? *12*. Were the basic data adequately described? *13*. Does the response rate raise concerns about non-response bias? *14*. If appropriate, was information about non-responders described? *15*. Were the results internally consistent? *16*. Were the results for the analyses described in the methods, presented? 17. Were the authors' discussions and conclusions justified by the results? *18*. Were the limitations of the study discussed? *19*. Were there any funding sources or conflicts of interest that may affect the authors' interpretation of the results? *20*. Was ethical approval or consent of participants attained?

References

Carson, V., Tremblay, M. S., Chaput, J.-P., & Chastin, S. F. M. (2016). Associations between sleep duration, sedentary time, physical activity, and health indicators among Canadian children and youth using compositional analyses. *Applied Physiology, Nutrition, and Metabolism, 41*(6 (Suppl. 3)), S294-S302. doi:10.1139/apnm-2016-0026

Carson, V., Tremblay, M. S., & Chastin, S. F. M. (2017). Cross-sectional associations between sleep duration, sedentary time, physical activity, and adiposity indicators among Canadian preschool-aged children using compositional analyses. *BMC Public Health, 17*(5), 848. doi:10.1186/s12889-017-4852-0

Chen, H., Wang, L.-j., Xin, F., Liang, G., & Zhou, Y.-l. (2023). Associations between 24-h movement behaviours and BMI in Chinese primary- and middle- school students. *Journal of Exercise Science & Fitness, 21*(2), 186-192. doi:https://doi.org/10.1016/j.jesf.2023.01.002

Domingues, S. F., Diniz da Silva, C., Faria, F. R., de Sá Souza, H., & dos Santos Amorim, P. R. (2022). Sleep, sedentary behavior, and physical activity in Brazilian adolescents: Achievement recommendations and BMI associations through compositional data analysis. *PLOS ONE, 17*(4), e0266926. doi:10.1371/journal.pone.0266926

Dumuid, D., Stanford, T. E., Martin-Fernández, J.-A., Pedišić, Ž., Maher, C. A., Lewis, L. K., . . . Olds, T. (2017). Compositional data analysis for physical activity, sedentary time and sleep research. *Statistical Methods in Medical Research, 27*(12), 3726-3738. doi:10.1177/0962280217710835

Fairclough, S. J., Dumuid, D., Taylor, S., Curry, W., McGrane, B., Stratton, G., . . . Olds, T. (2017). Fitness, fatness and the reallocation of time between children's daily movement behaviours: an analysis of compositional data. *International Journal of Behavioral Nutrition and Physical Activity, 14*(1), 64. doi:10.1186/s12966-017-0521-z

Haszard, J. J., Meredith-Jones, K., Farmer, V., Williams, S., Galland, B., & Taylor, R. (2020). Non-Wear Time and Presentation of Compositional 24-Hour Time-Use Analyses Influence Conclusions About Sleep and Body Mass Index in Children. *Journal for the Measurement of Physical Behaviour, 3*(3), 204-210. doi:10.1123/jmpb.2019-0048

Healy, S., Brewer, B., Garcia, J., Daly, J., & Patterson, F. (2021). Sweat, Sit, Sleep: A Compositional Analysis of 24-hr Movement Behaviors and Body Mass Index among Children with Autism Spectrum Disorder. *Autism Research, 14*(3), 545-550. doi:https://doi.org/10.1002/aur.2434

Kuzik, N., Naylor, P.-J., Spence, J. C., & Carson, V. (2020). Movement behaviours and physical, cognitive, and social-emotional development in preschool-aged children: Cross-sectional associations using compositional analyses. *PLOS ONE, 15*(8), e0237945. doi:10.1371/journal.pone.0237945

Lund Rasmussen, C., Gába, A., Stanford, T., Dygrýn, J., Dumuid, D., Janda, D., & Hron, K. (2023). The Goldilocks Day for healthy adiposity measures among children and adolescents. *Frontiers in Public Health, 11*. doi:10.3389/fpubh.2023.1158634

McGee, M., Unger, S., Hamilton, J., Birken, C. S., Pausova, Z., Vanderloo, L. M., . . . O'Connor, D. L. (2020). Lean mass accretion in children born very low birth weight is significantly associated with estimated changes from sedentary time to light physical activity. *Pediatric Obesity, 15*(5), e12610. doi:https://doi.org/10.1111/ijpo.12610

Migueles, J. H., Delisle Nyström, C., Dumuid, D., Leppänen, M. H., Henriksson, P., & Löf, M. (2023). Longitudinal associations of movement behaviours with body composition and physical fitness from 4 to 9 years of age: structural equation and mediation analysis with compositional data. *International Journal of Behavioral Nutrition and Physical Activity, 20*(1), 11. doi:10.1186/s12966-023-01417-1

Ng, E., Wake, M., Olds, T., Lycett, K., Edwards, B., Le, H., & Dumuid, D. (2021). Equivalence Curves for Healthy Lifestyle Choices. *Pediatrics, 147*(4), e2020025395. doi:10.1542/peds.2020-025395

Talarico, R., & Janssen, I. (2018). Compositional associations of time spent in sleep, sedentary behavior and physical activity with obesity measures in children. *International Journal of Obesity, 42*(8), 1508-1514. doi:10.1038/s41366-018-0053-x

Taylor, R. W., Haszard, J. J., Meredith-Jones, K. A., Galland, B. C., Heath, A.-L. M., Lawrence, J., . . . Taylor, B. J. (2018). 24-h movement behaviors from infancy to preschool: cross-sectional and longitudinal relationships with body composition and bone health. *International Journal of Behavioral Nutrition and Physical Activity, 15*(1), 118. doi:10.1186/s12966-018-0753-6

Zhang, T., Li, H., Li, C., Zhang, L., & Zhang, Z. (2022). The Compositional Impacts of 2 Distinct 24-Hour Movement Behavior Change Patterns on Physical Fitness in Chinese Adolescents. *Journal of Physical Activity and Health, 19*(4), 284-291. doi:10.1123/jpah.2021-0778
